# Leakage-Controlled and Information-Bounded Evaluation of Multi-Task Learning for Crumb–Rubber Modified Asphalt: What Twenty Mix Designs Can and Cannot Support

**DOI:** 10.3390/ma19173727

**Published:** 2026-09-01

**Authors:** He Huang, Yingli Gao, Bin Tian, Zhuo Yang

**Affiliations:** 1 School of Transportation, Changsha University of Science and Technology, Changsha 410114, China; huanghe_r@163.com (H.H.);; 2 Hunan Provincial Engineering Technology Research Center for Novel and Carbon Neutral Road Material, Changsha University of Science and Technology, Changsha 410114, China

**Keywords:** crumb–rubber modified asphalt, mix design-grouped cross-validation, nested model selection, information ceiling, multi-task learning, mixture of experts, small-sample evaluation

## Abstract

Data-driven prediction of crumb–rubber modified asphalt (CRMA) binder properties is usually reported on datasets in which a small number of mix-designs is swept across several test temperatures, so that the row count greatly exceeds the number of independent experiments. This study asks what such a dataset can actually support. Using 221 laboratory measurements drawn from 20 independent CRMA mix designs, we evaluate a task-adaptive mixture-of-experts multi-task network (TA-MoE-MTL), a locked Huber-anchored hybrid extension, and fourteen deep and classical reference models for the joint prediction of penetration, softening point, ductility and rutting factor. Three methodological elements are introduced. First, model selection is made strictly nested and group-aware: the stopping epoch is chosen on an inner split of the training designs and the held-out designs are used once. Second, we bound what the recorded inputs can explain before any model is fitted: because the consistency targets are constant within a mix design and because eight designs share identical input vectors while their rutting factors differ, the attainable coefficient of determination for the rutting factor is 0.790 rather than unity. Third, performance is reported with each mix design weighted equally, so that high replication designs cannot dominate. The locked hybrid assigns 90% weight to a Huber-anchored robust expert branch and 10% to a freshly trained TA-MoE-MTL branch. It attains pooled out-of-fold coefficients of determination of 0.613/0.594/0.878/0.685 and ranks first of 16 models at a mean pooled R^2^ 0.693, exceeding MLP–sklearn (0.656) by 0.037. Across three seeds, seeds 42/43/44 give mean pooled R^2^ values of 0.693/0.689/0.693 (mean 0.691 ± 0.002), and all three runs remain above the frozen MLP sklearn reference. A learning curve over the number of training designs is still rising at the largest size the data allow. The contribution of this work is an evaluation protocol for replicated mix design datasets, a way of bounding their information content, and a robust hybrid that exposes rather than hides the value of a simple small-sample anchor.

## 1. Introduction

Crumb–rubber modified asphalt is an established route to high-performance pavement binders and to the valorisation of waste tyre rubber [1,2]. Its engineering behaviour is summarised by a small set of standard properties—penetration, softening point, ductility and the Superpave rutting factor—that emerge from the coupled influence of base bitumen chemistry, rubber dosage, particle size and activation route, warm mix additive chemistry, and the thermo-mechanical history imposed during blending [3]. Because laboratory characterisation of each candidate mix is slow and expensive, predicting these properties from the mix-design is an attractive target for machine learning, and a substantial literature now reports high accuracies for exactly this task [4,5,6,7,8], including multi-task formulations that predict several pavement properties jointly [9].

That literature shares a structural feature that has received little attention. Datasets in this field are typically assembled by preparing a modest number of binder formulations and then measuring each of them repeatedly under a sweep of test temperatures [10,11]. The resulting table has many rows, but the number of independent experiments is the number of formulations, which is often an order of magnitude smaller. When such a table is split at random, temperature replicates of the same physical binder appear on both sides of the split, and the model is rewarded for recognising the formulation rather than for predicting its behaviour. The dataset analysed here is a concrete instance: 221 measurements correspond to 20 independent mix designs.

Grouping the cross-validation folds by mix design removes that leakage path, as in a previous version of this work. Two further problems, however, survive grouping, and both are addressed here for the first time. The first is that model selection can reintroduce the leakage that the split removed: if the stopping epoch of a deep network is chosen by monitoring the same held-out designs that are later used to report performance, and if there are only three to five such designs, the reported number is optimistic by an amount that has never been measured in this setting. Second, the recorded inputs may not determine the recorded outputs at all. If two measurements share an identical input vector but differ in their target, no deterministic model can separate them, and the residual is not a modelling failure but a property of the data.

Both problems are quantitative rather than rhetorical, and both can be measured. This paper measures them. We retrain every configuration under nested, group-aware model selection and report the difference from the common protocol; before fitting anything, we compute the largest coefficient of determination that any deterministic function of the recorded inputs could achieve on each target. Only then do we compare architectures.

### Research Gap and Contribution

Three limitations of the existing CRMA machine learning literature motivate this study. First, replicate structure is rarely controlled: reported accuracies are usually obtained under random splitting of measurements that are not independent, so they answer a question about recognising known formulations rather than about predicting new ones. Second, the attainable accuracy of a dataset is never established before models are compared, so a model that recovers most of the available signal is indistinguishable from one that recovers little. Third, model selection is routinely performed on the fold used to report performance, which is a leakage path that grouping alone does not close.

Against each of these, we contribute the following. (i) A complete evaluation protocol for replicated mix-design datasets, in which the folds are grouped by mix design, all preprocessing is fitted inside the training fold, and the stopping epoch is chosen on an inner group-disjoint split; the optimistic bias of the usual alternative is measured at +0.244 (range +0.024 to +0.412 over the eight headline models) in mean coefficient of determination. (ii) An information ceiling analysis that bounds, per target, what the recorded inputs can explain, together with the finding that the ceiling for the rutting factor in this dataset is 0.790 and that three of the four targets carry far less information than their row count suggests. (iii) A benchmark of sixteen model configurations under this protocol, with recipe-balanced metrics, recipe-level bootstrap effect sizes, leave-one-recipe-out validation, and a learning curve over the number of training designs. (iv) A locked hybrid in which a robust Huber anchor carries 90% of the prediction and a freshly trained TA-MoE-MTL branch carries 10%; it ranks first of 16 models at a mean pooled R^2^ of 0.693, exceeding MLP–sklearn (0.656) by 0.037, while task-wise R^2^ differences from MLP–sklearn are −0.009/−0.049/−0.006/+0.211; none of the four differences survives Holm correction, and all four 95% recipe bootstrap intervals include zero.

## 2. Dataset and Its Structure

### 2.1. Measurements and Variables

The dataset consolidates 221 valid measurements from in-house CRMA mixing campaigns. Thirteen process variables are recorded for each measurement: shear time and shear speed; the penetration, softening point and ductility of the base bitumen; rubber dosage, particle size and activation method; warm mix additive dosage and type; and three test condition temperatures, namely the base bitumen ductility test temperature, the DSR test temperature and the ductility test temperature. Four properties are predicted. Ductility is measured for 155 of the 221 rows and is handled by an explicit task mask in both the objective and the metrics.

The distinction between the ten mix-design variables and the three test condition temperatures is central to everything that follows. The former describe the material; the latter describe the measurement applied to it after it was produced. Only the mix-design variables define an independent experiment.

### 2.2. Twenty Independent Mix Designs

Grouping the measurements by the ten mix-design variables yields 20 distinct designs, identified throughout as R01 to R20. These are transparent analytical identifiers for the exact unique ten-variable combinations in the deposited workbook; the workbook contains no independent batch identifier with which to verify physical batch identity. A precision audit shows that the exact values and every rounding precision from two to eight decimal places all yield 20 groups, so the count is not an artefact of the six-decimal implementation choice. Table 1 lists the groups in full, with the replication count and the test temperatures applied, and the mean of each target. Replication is strongly unequal: designs contribute between four and twenty-three measurements each, and only eight of the twenty carry ductility.

**Figure 1:**
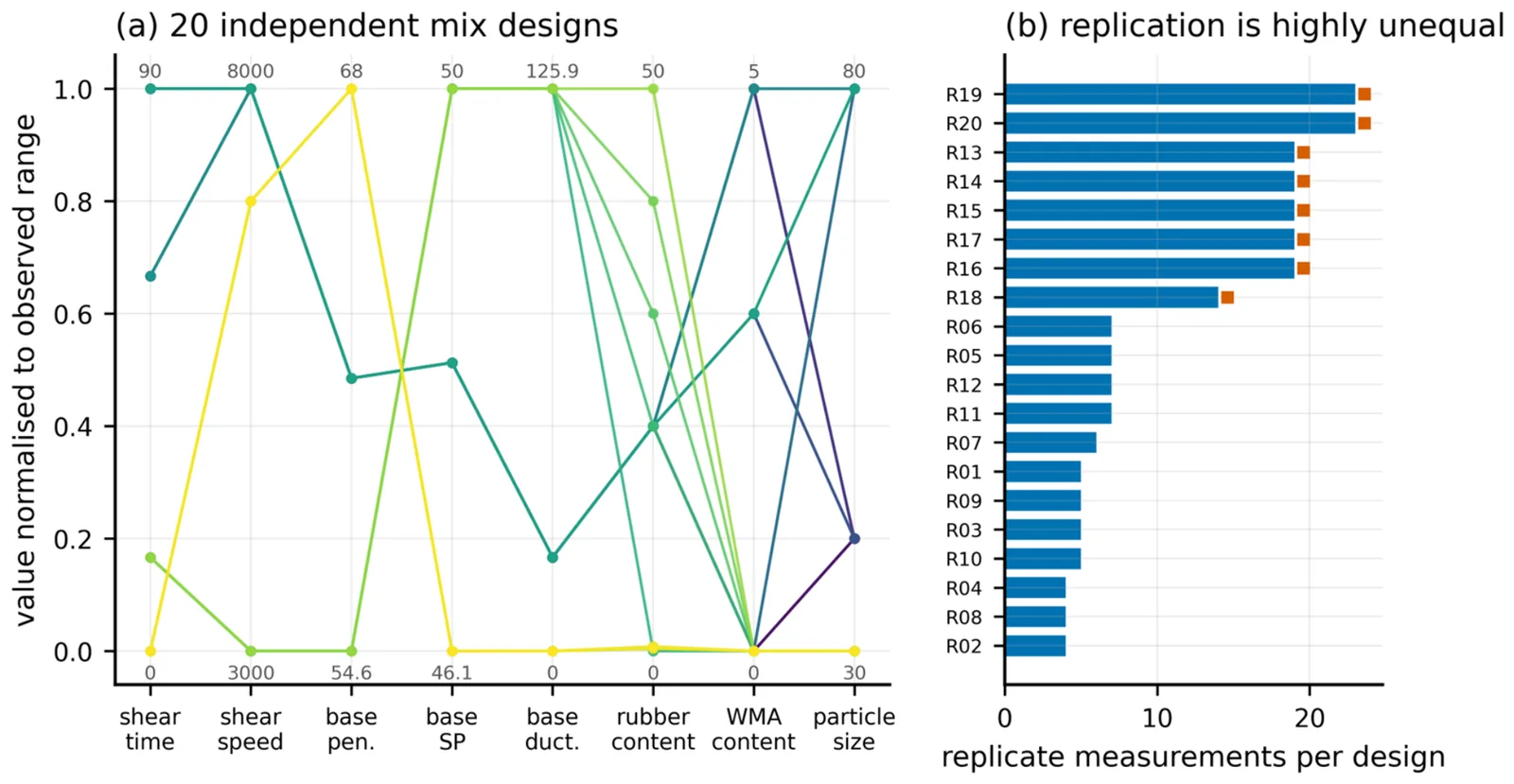
Coverage of the mix-design space by the 20 independent designs. (**a**) Each line is one design, plotted across the eight numeric mix-design variables normalised to their observed range; the extreme values of each variable are annotated. (**b**) Replicate count per design; squares mark the 8 designs that carry ductility measurements.

Figure 1 shows why the row count is misleading. Several variables take only two or three distinct values across the entire campaign, and the designs cluster into a small number of families rather than sampling the space. A model trained on this table can interpolate between neighbouring designs; it has no basis for extrapolating to a formulation family that the campaign did not visit.

### 2.3. Physics-Informed Feature Engineering

Thirteen derived variables extend the raw process variables to a 26-dimensional input. Table 2 gives each definition together with the mechanism it is intended to encode and the assumption that makes the encoding meaningful. Two points should be read alongside that table. The derived variables are deterministic functions of the raw ones, so they add no information; their purpose is to make plausible interaction terms available to models that would otherwise have to learn them. And they introduce substantial redundancy: 41 of the input pairs have an absolute Pearson correlation of at least 0.85. Attributions are therefore aggregated by physical factor in Section 4.4 rather than read column by column.

### 2.4. What These Data Can Support

**Figure 2:**
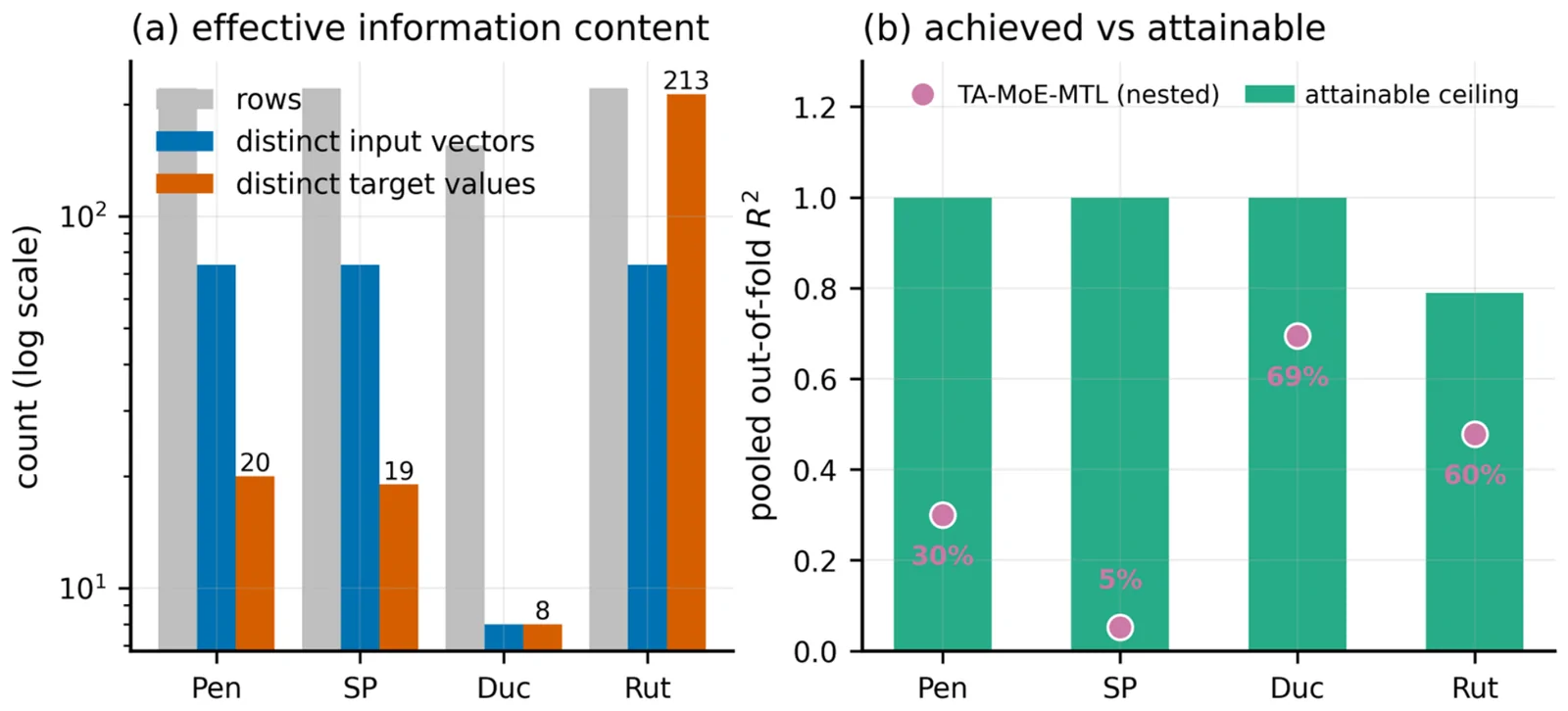
What the data can support. (**a**) For each target, the number of rows, the number of distinct input vectors, and the number of distinct target values, on a logarithmic scale. (**b**) The attainable coefficient of determination from Equation (1) compared with the value achieved by TA-MoE-MTL under the nested protocol; labels give the achieved fraction of the ceiling.

Before any model is fitted, it is worth asking how much of each target the recorded inputs could explain in principle. Let the input vectors be partitioned into classes of identical value. The best possible deterministic predictor assigns to every member of a class the class mean of the target, so the largest attainable coefficient of determination is

(1) $$R_{max}^{2}=1-\frac{\sum_{c\in C}\sum_{i\in c}{\left(y_{i}-{\bar{y}}_{c}\right)}^{2}}{\sum_{i}{\left(y_{i}-\bar{y}\right)}^{2}}$$

which is one when the inputs identify the target and less than one when they do not. Table 3 reports this quantity for the four targets and Figure 2 places the achieved performance against it. Three consequences follow, and each of them is a property of the data rather than of any model.

First, the three consistency targets have no within-design variation. Penetration, softening point, and ductility are recorded once per mix design and repeated across that design’s temperature replicates. Across the whole table, they therefore take 20, 19, and 8 distinct values, respectively. The attainable coefficient of determination for these three targets is one, but the effective sample size for fitting them is the number of designs and, for ductility, only eight designs and eight distinct numbers. Any accuracy reported for ductility on 155 rows should be read with that in mind, and we report it separately for that reason.

Second, the rutting factor is not identified by the recorded inputs. Eight mix designs, covering 155 of the 221 rows, have byte-identical 26-dimensional input vectors while their measured G*/sin δ values span up to three orders of magnitude within a single design. The recorded DSR test temperature does not vary with the sweep temperature actually applied to those specimens. 21.0% of the rutting factor variance is therefore unreachable by any deterministic function of these inputs, and the ceiling is 0.790. This is a limitation of how the campaign was recorded, and correcting it in future campaigns is the single most valuable change available to this line of work.

Third, the rubber dosage column carries two unit conventions. Designs R18, R19 and R20 record the dosage as 0.2, 0.3 and 0.4, whereas the remaining designs record values between 20 and 50, and the penetrations measured for R18 to R20 are not consistent with a sub-one-per-cent dosage. We have not altered the archived measurements. All results in this paper use the values exactly as recorded, and Section 4.3 reports a unit-harmonised sensitivity run in which the three designs are rescaled by a factor of one hundred: as recorded 0.300/0.052/0.695/0.478 versus unit-harmonised 0.382/0.279/0.739/0.300 (mean R^2^ 0.381 versus 0.425; largest per-task change +0.227). The conclusions do not depend on the resolution of this ambiguity, but readers of the deposited data should be aware of it.

## 3. Model

This section defines the architecture that is evaluated. It contains no experimental results; implementation details are given in Supplementary Section S2.

### 3.1. Overview

TA-MoE-MTL predicts all four targets from one shared encoder, following the mixture-of-experts principle of decoupling capacity from specialisation through gated routing [12,13,14] and its recent adaptations to tabular and multi-task settings [15,16]. The encoder embeds each input variable as a token, passes the token sequence through four experts with different inductive biases, and mixes the expert representations with a gate that is learned separately for each target. Three auxiliary components complete the model: a target normaliser that log-transforms heavily skewed targets using statistics computed inside the training fold only; a distillation term that pulls the weaker experts towards the best-performing one at each optimisation step; and a homoscedastic uncertainty weighting that balances the four task losses.

### 3.2. Log-Aware Target Normalisation

Heavily skewed regression targets are known to destabilise gradient-based training [17]. For each target, the sample skewness is computed on the training fold. Targets whose skewness exceeds a threshold tau are log-transformed with the transformation log(1 + y); all targets are then standardised. Both the decision and the standardisation statistics come from the training fold alone, and the inverse transformation is applied before any metric is computed. The value of tau is a preprocessing choice and not a tuned parameter; Section 4.3 reports the sensitivity of the results to it.

(2) $$y\prime_{t}=\begin{array}{ll} log\left(1+y_{t}\right), & \mathrm{if}skew\left(y_{t}\mathrm{on}\;\mathrm{the}\;\mathrm{training}\;\mathrm{fold}\right)>\tau ,\\ y_{t}, & \mathrm{otherwise}. \end{array}$$

(3) $$z_{i,t}=\frac{y\prime_{i,t}-\mu_{t}}{\nu_{t}},\;\;\mu_{t}\;\mathrm{and}\;\nu_{t}\;\mathrm{estimated}\;\mathrm{from}\;\mathrm{the}\;\mathrm{training}\;\mathrm{fold}$$

(4) $${\hat{y}}_{i,t}=\begin{array}{ll} exp\left(\nu_{t}{\hat{z}}_{i,t}+\mu_{t}\right)-1, & \mathrm{for}\;a\;\mathrm{logged}\;\mathrm{target},\\ \nu_{t}{\hat{z}}_{i,t}+\mu_{t}, & \mathrm{otherwise}. \end{array}$$

Equations (2)–(4) make the leakage boundary explicit. The log flags, mu_t and nu_t, are recomputed for every outer and inner training split. Predictions are returned to physical units before clipping and before R^2^, RMSE, or MAE is evaluated.

### 3.3. Task-Adaptive Mixture of Experts

Each of the d input variables is mapped to a learned embedding by its own linear projection, and the embeddings are stacked into a sequence of d tokens. Four experts consume this sequence: a long short-term memory network [18], a gated recurrent unit, a selective state space block [19], and a self-attention block [20,21]. The sequence architectures are used here as tabular encoders rather than as time series models. They are not inherently invariant to the arbitrary feature order; Section 4.3 quantifies that sensitivity and treats it as a limitation. In the implementation, d = 26, each token has dimension D = 64, and each expert returns one D-dimensional vector per measurement.

(5) $$h_{j}=W_{j}x_{j}+b_{j}+p_{j},\;j=1,\ldots ,d;\;\;H=\left[h_{1};h_{2};\ldots ;h_{d}\right]$$

Here W_j and b_j are feature-specific projection parameters, and p_j is a learned feature-position embedding. Equation (5) turns each scalar engineered variable into a token while retaining its identity.

(6) $$LSTM:\;c_{j}=f_{j}⊙c_{j-1}+i_{j}⊙g_{j},\;q_{j}=o_{j}⊙tanh\left(c_{j}\right),\;z_{1}=\mathrm{LN}\;\left(W_{L}{mean}_{j}\left(q_{j}\right)\right)$$

(7) $$GRU:\;q_{j}=\left(1-u_{j}\right)⊙q_{j-1}+u_{j}⊙{\tilde{q}}_{j},\;z_{2}=\mathrm{LN}\;\left(W_{G}{mean}_{j}\left(q_{j}\right)\right)$$

(8) $$Mamba:\;s_{j}={\bar{A}}_{j}s_{j-1}+{\bar{B}}_{j}h_{j},\;q_{j}=C_{j}s_{j}+D_{m}h_{j},\;z_{3}=\mathrm{LN}\;\left({mean}_{j}\left(q_{j}\right)\right)$$

(9) $$\begin{array}{l} Attention\left(H\right)=softmax\;\left(\frac{QK^{⊤}}{\sqrt{d_{k}}}\right)V,\;Q=HW_{Q},\;K=HW_{K},\;V=HW_{V};\\ z_{4}=\mathrm{LN}\;\left({mean}_{j}(Transformer{\left(H\right)}_{j})\right) \end{array}$$

Equations (6)–(9) summarise the four expert maps. The LSTM and GRU use two bidirectional recurrent layers; the lightweight selective state space expert uses two residual Mamba-style blocks; and the AutoDLFormer expert uses three multi-head self-attention layers. The operator .* denotes elementwise multiplication. Layer normalisation places all four outputs on a comparable scale before routing.

(10) $$Z=\left[z_{1};z_{2};z_{3};z_{4}\right]\in R^{E\times D},\;\;E=4,\;D=64$$

For each target, a two-layer gate consumes the standardised engineered input x_i and produces a distribution over the four experts. This matches the released implementation: the gate is conditioned on the input vector directly, rather than on the pooled expert outputs.

(11) $$a_{i,t}=softmax\;\left(\frac{G_{t}\left(x_{i}\right)}{\kappa_{g}}\right),\;\;\sum_{e}a_{i,t,e}=1,\;a_{i,t,e}\geq 0$$

(12) $$u_{i,t}=\sum_{e=1}^{E}a_{i,t,e}z_{i,e}$$

(13) $${\hat{z}}_{i,t}={Head}_{t}\left(u_{i,t}\right)$$

The penetration head contains two hidden transformations because it was the most difficult consistency target during development; the other three heads contain one. All heads output in LATN-normalised target space, and Equation (4) maps their predictions back to the measurement unit.

### 3.4. Cross-Expert Distillation and the Objective

Knowledge distillation across the heads of a multi-task network is an established way of sharing information between related tasks [22,23]; here the same idea is applied inside the mixture, between experts. At every optimisation step and for every target, the expert with the lowest task loss on the current batch is designated the teacher, and the remaining experts are pulled towards its predictions. Section 4.2 reports how often that designation changes, which determines how this term should be interpreted. Let m_(i,t) be one only when target t is observed for row i.

(14) $$L_{t}=\frac{\sum_{i}m_{i,t}{\left({\hat{z}}_{i,t}-z_{i,t}\right)}^{2}}{\sum_{i}m_{i,t}+\varepsilon }$$

(15) $$e_{t}^{teacher}=arg\underset{e}{min}\left\{\frac{\sum_{i}m_{i,t}{\left({\hat{z}}_{i,e,t}-z_{i,t}\right)}^{2}}{\sum_{i}m_{i,t}+\varepsilon }\right\}$$

(16) $$L_{CEKD}={mean}_{i,t,e\neq e_{t}^{teacher}}\left\{m_{i,t}{\left[\frac{{\hat{z}}_{i,e,t}-stopgrad\left({\hat{z}}_{i,e_{t}^{teacher},t}\right)}{\kappa_{d}}\right]}^{2}\right\}$$

(17) $$L_{total}=\sum_{t=1}^{T}\left[exp\left(-s_{t}\right)L_{t}+s_{t}\right]+\lambda L_{CEKD}$$

Equation (14) prevents unmeasured ductility labels from entering the gradient. Equation (15) selects the lowest-loss auxiliary expert on the current mini-batch and task. The teacher prediction is detached in Equation (16), so CEKD moves the other auxiliary heads without back-propagating through the selected teacher. In Equation (17), s_t is a learnable log variance implementing homoscedastic task weighting [24], lambda is the distillation weight, and kappa_d is the distillation temperature.

### 3.5. Huber-Anchored Hybrid Extension

The corrected experiments show that the high-capacity neural model and its CEKD objective are not uniformly beneficial with only twenty mix designs. We therefore add a robust expert bank whose convex gate is regularised towards a Huber regression expert, and lock the final prediction rule before confirmation: 90% of each target prediction comes from this Huber-anchored branch, and 10% from a TA-MoE-MTL branch retrained inside the same outer fold. The Huber expert, all feature scaling, and the neural stopping epoch are fitted using only the outer training designs. The weights are fixed rather than tuned on the reported fold. This model is denoted Huber-anchored Hybrid TA-MoE-MTL. The original TA-MoE-MTL remains in all tables as the mechanism and ablation reference. For target t, the anchor expert minimises Huber loss in the same fold local normalised target space.

(18) $$\rho_{\delta }\left(r\right)=\begin{array}{ll} 1/2r^{2}, & \left|r\right|\leq \delta ,\\ \delta \left(\left|r\right|-1/2\delta \right), & \left|r\right|>\delta . \end{array}$$

(19) $$\alpha_{t}^{⋆}=arg\underset{\alpha \in \Delta }{min}\left\{\frac{{MSE}_{w}\left(y_{t},P_{t}\alpha \right)}{{Var}_{w}\left(y_{t}\right)}+\gamma ∥\alpha -e_{H}{∥}_{2}^{2}+\eta ∥\alpha {∥}_{2}^{2}\right\}$$

(20) $${\hat{y}}_{i,t}^{locked}=0.90\;{\hat{y}}_{i,t}^{\text{Huber-anchor}}+0.10\;{\hat{y}}_{i,t}^{\text{TA-MoE-MTL}}$$

The residual r in Equation (18) is the difference between the Huber prediction and the observed normalised target, with delta = 1.35. Quadratic treatment near zero preserves efficiency, while linear growth outside delta limits the influence of extreme rows. In Equation (19), P_t contains inner recipe-disjoint OOF predictions from the robust expert bank; w gives every recipe equal total mass; e_H selects the Huber expert; gamma = 10; and eta = 0.001. This variance-normalised simplex problem makes the regularisation comparable across targets with different units. Equation (20) is not fitted on the reported fold: both branches are trained using only its outer training designs, the 0.90/0.10 weights are fixed, and the outer fold is evaluated once. This explicit formula is also the configuration used in the three-seed, reduced-input and LORO confirmations.

## 4. Evaluation and Results

### 4.1. Evaluation Protocol and Metrics

Measurements are partitioned into five folds by mix design, so that all replicates of a design fall on the same side of every split. Table 4 gives the composition of each fold. Two features of that table deserve emphasis, because they limit what fold-level statistics can mean here: two of the five folds receive ductility measurements from a single design each, so the within-fold ductility variance is exactly zero and a fold-level coefficient of determination is undefined; and the fold-mean rutting factor ranges from 12 kPa to 1013 kPa.

Every preprocessing step is fitted inside the training fold: the log-transformation decision and its statistics, the input standardiser, the prediction range used for clipping, and the hyper-parameters of the classical baselines. Deep models select their stopping epoch on an inner group-disjoint split holding out a quarter of the training designs, and are then refit on the complete training fold for the selected number of epochs. The held-out fold is used exactly once to generate predictions. We refer to this as the nested protocol.

**Figure 3:**
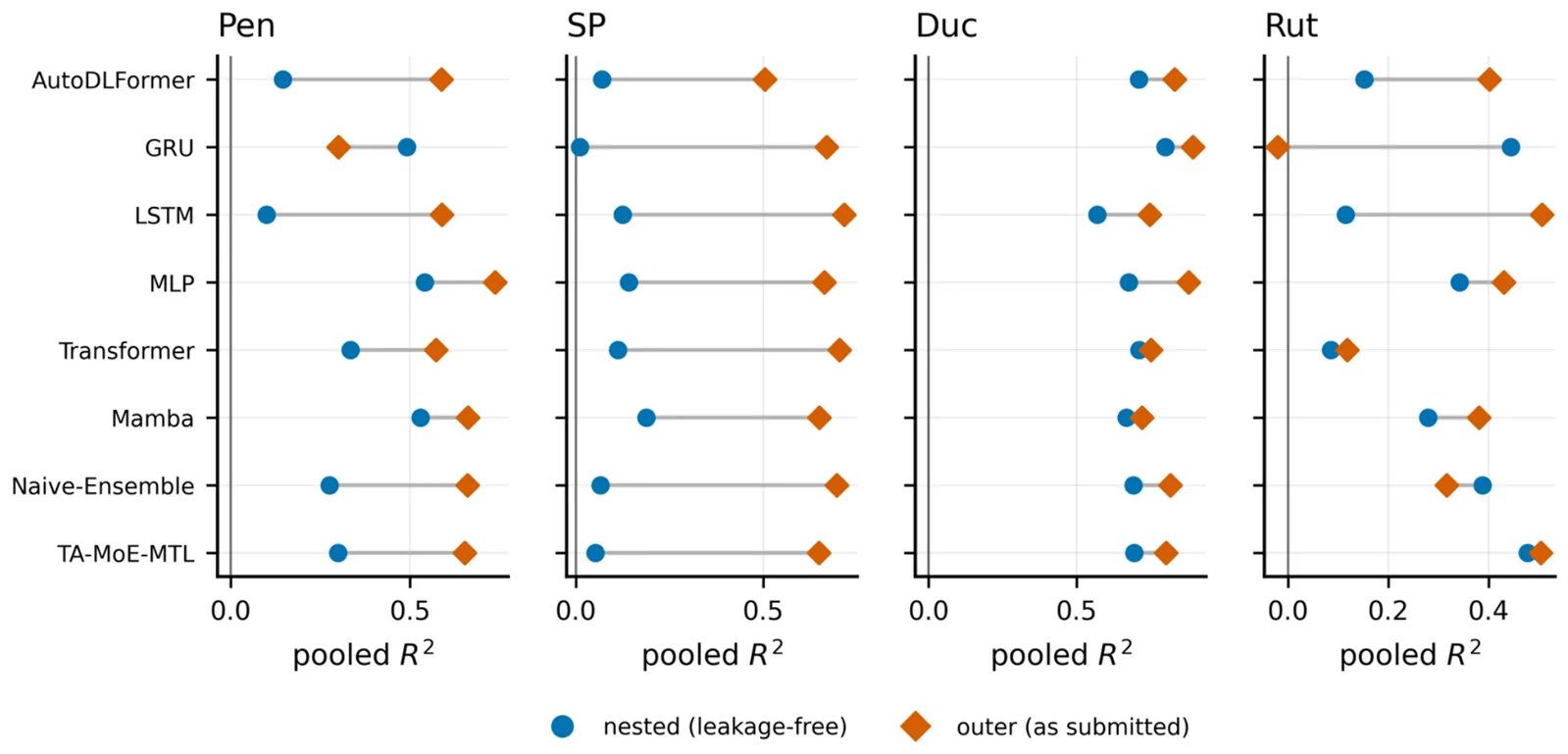
Optimistic bias of selecting the stopping epoch on the reported fold. Each pair of markers is one model; the circle is the nested protocol and the diamond is the protocol used in the submitted version.

To measure what the more common alternative is worth, we also rerun the eight headline models under the protocol used in the submitted version of this work, in which the stopping epoch is chosen by monitoring the held-out fold itself. The difference is reported in Table 5 and Figure 3 and is +0.244 (range +0.024 to +0.412 over the eight headline models) in the mean pooled coefficient of determination. Per model: AutoDLFormer +0.312; GRU +0.024; LSTM +0.412; MLP +0.252; Transformer +0.225; Mamba +0.187; Naive-Ensemble +0.267; TA-MoE-MTL +0.271. The bias is not constant across architectures, so a comparison under that protocol is unsafe: it rewards models with the most capacity to fit whatever is being monitored. A uniform offset would still leave the ordering of models usable; this one does not, because the two protocols rank the eight headline models with a Spearman correlation of −0.02; MLP is first under the submitted protocol but two of eight under the nested one, where GRU is first, and GRU moves seven places.

Two coefficients of determination are reported for every model and target. The pooled value is computed on the concatenation of the five sets of held-out predictions and weights each measurement equally, so designs with twenty-three replicates influence it roughly six times as much as designs with four. The recipe-balanced value weights each mix design equally and is the quantity aligned with the stated objective of generalising to unseen designs. Root mean square and mean absolute errors are given in physical units in the supplement. For the proposed model, the two coefficients are pooled 0.300/0.052/0.695/0.478 versus recipe-balanced 0.107/0.093/0.710/0.528.

### 4.2. Main Results and Component Evidence

**Figure 4:**
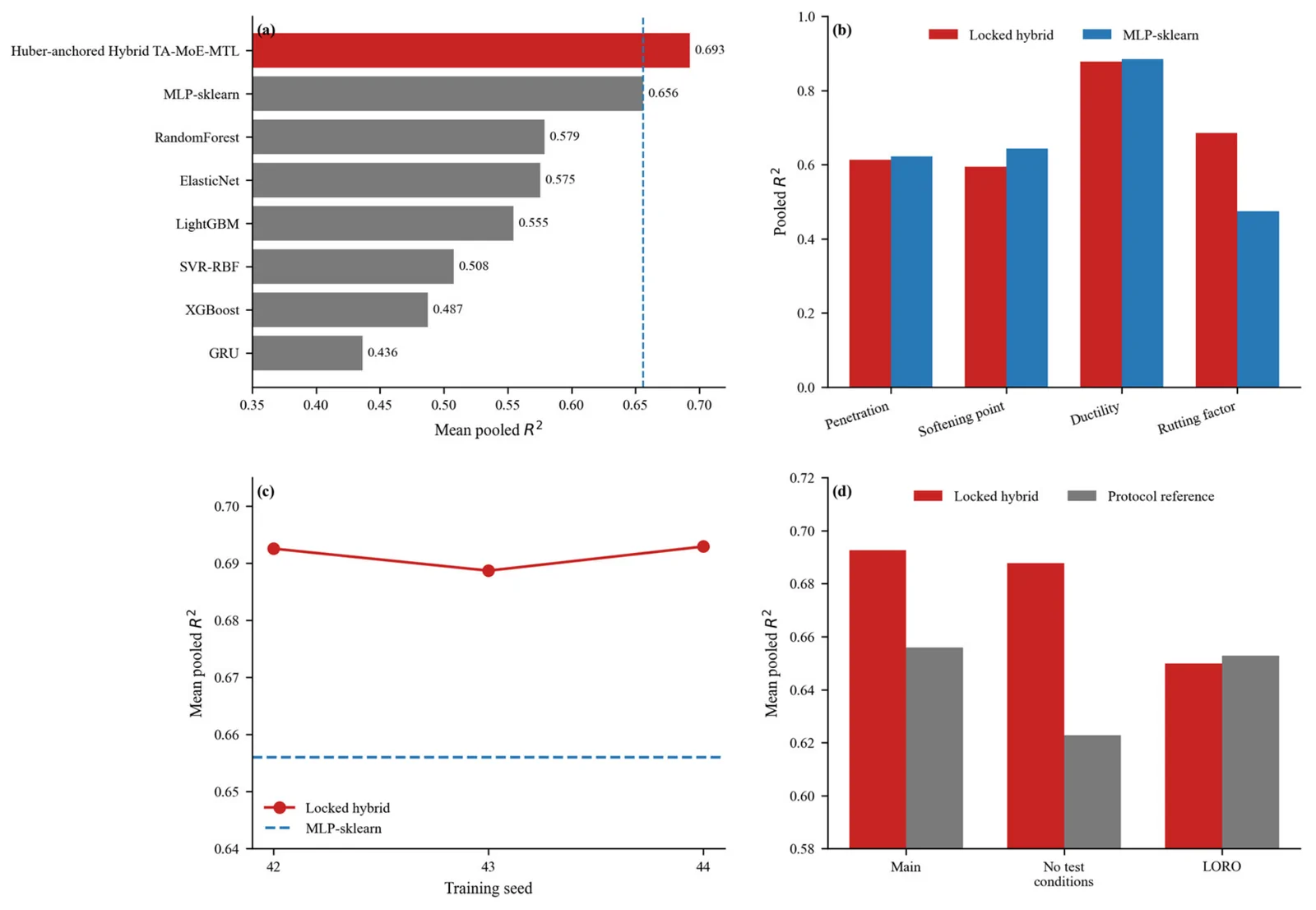
Validation of the locked Huber-anchored Hybrid TA-MoE-MTL. (**a**) Overall ranking; (**b**) task-wise comparison with MLP–sklearn; (**c**) three training seeds; and (**d**) the main, no-test condition and LORO settings. The LORO panel is retained although the locked hybrid is slightly below the corresponding reference.

Sixteen configurations were evaluated: the locked hybrid, the original TA-MoE-MTL, seven other deep configurations, and seven classical regressors. Table 6 and Figure 4 report the same held-out predictions. The locked hybrid attains pooled R^2^ values of 0.613/0.594/0.878/0.685 for penetration, softening point, ductility, and rutting factor; it ranks first of 16 models at a mean pooled R2 of 0.693, exceeding MLP–sklearn (0.656) by 0.037. Its recipe-balanced mean R^2^ is 0.764, and the rutting score reaches 87% of the recorded input ceiling.

The improvement is deliberately attributed to robust anchoring, not to the original adaptive gate: the locked prediction rule assigns 90% to a Huber-anchored robust expert branch and 10% to a freshly trained TA-MoE-MTL branch in every outer fold. The final model is slightly below MLP–sklearn on the three consistency targets and gains on the rutting factor. Accordingly, task-wise R^2^ differences from MLP–sklearn are −0.009/−0.049/−0.006/+0.211; none of the four survives Holm correction, and all four 95% recipe bootstrap intervals include zero. The result supports first place under the declared mean metric, but not a claim of uniform task-wise or statistically resolved dominance.

A single deterministic run is not enough to establish an ordering at this sample size. For the locked hybrid, seeds 42/43/44 give mean pooled R^2^ values of 0.693/0.689/0.693 (mean 0.691 ± 0.002), and all three runs remain above the frozen MLP–sklearn reference. For the original neural configurations, across five seeds, TA-MoE-MTL achieves 0.351 ± 0.037 (range 0.309–0.384); MLP achieves 0.287 ± 0.086 (range 0.219–0.425); and w-o-CEKD achieves 0.375 ± 0.098 (range 0.258–0.478). A second control performs fold-local inner selection over learning rate, weight decay, and exponential moving average settings for all eight deep architectures: TA-MoE-MTL reaches mean pooled R^2^ 0.279 and ranks fifth of eight, while GRU is first at 0.375; the selected TA-MoE-MTL setting changes across folds, and its inner score is negative in two of five folds. These controls change the ordering but not the conclusion: the available designs do not support a stable claim of architectural superiority.

**Figure 5:**
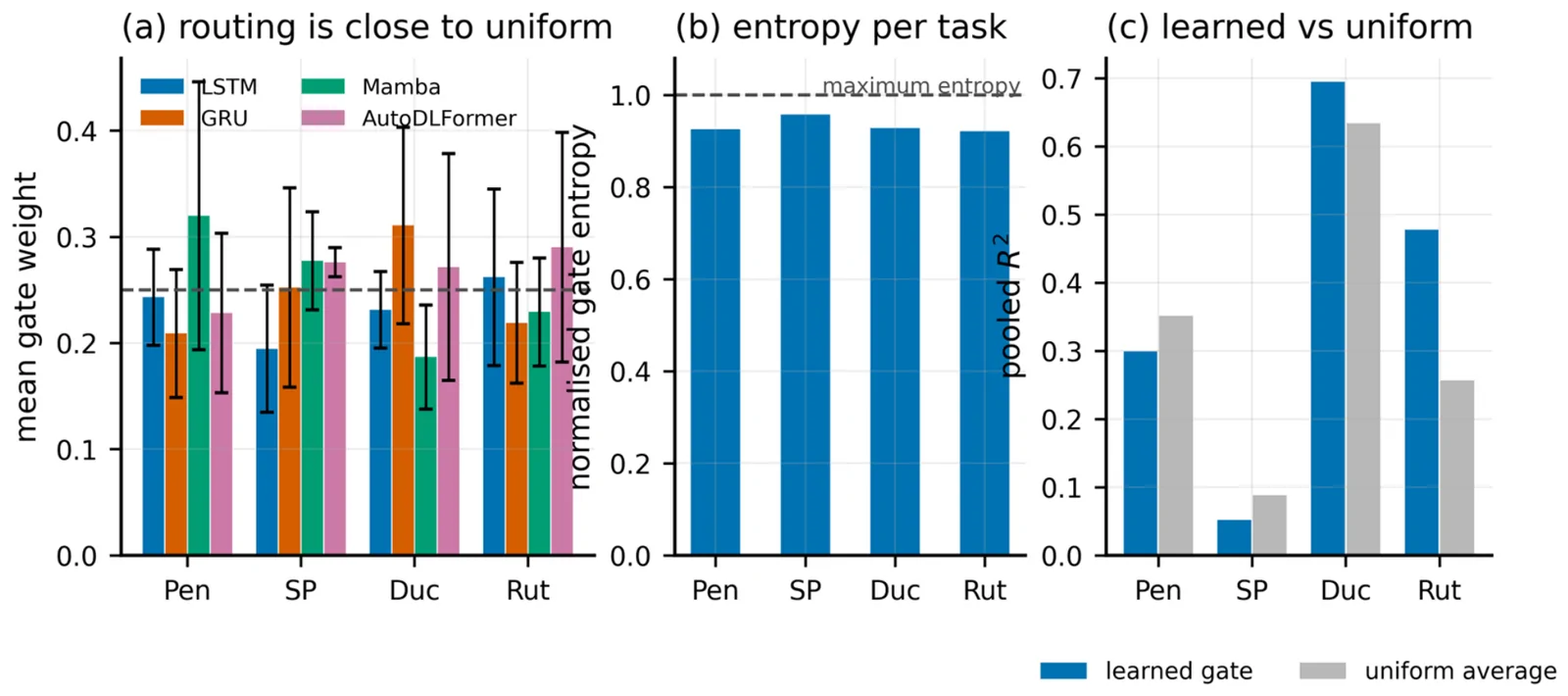
Behaviour of the task-adaptive gate. (**a**) Mean gate weight per target and expert, with error bars showing the spread across the five folds; the dashed line is uniform routing. (**b**) Normalised gate entropy per target. (**c**) Pooled coefficient of determination with the learned gate and with the gate replaced by a uniform average.

Mechanism diagnostics for the original neural branch. The submitted version attributed the model’s behaviour to task-adaptive routing. The diagnostics do not support that reading(see Figure 5): the normalised gate entropy is 0.925/0.957/0.928/0.922 for the four tasks, where 1.0 denotes uniform routing; individual weights span 0.19 to 0.32, and replacing the learned gate with a uniform average changes the mean pooled R^2^ from 0.381 to 0.333. A normalised entropy near one means that the gate distributes each target across all four experts almost evenly; the lowest value observed across the four targets is 0.922. Whatever the architecture is doing, it is not sharp, task-dependent routing.

**Figure 6:**
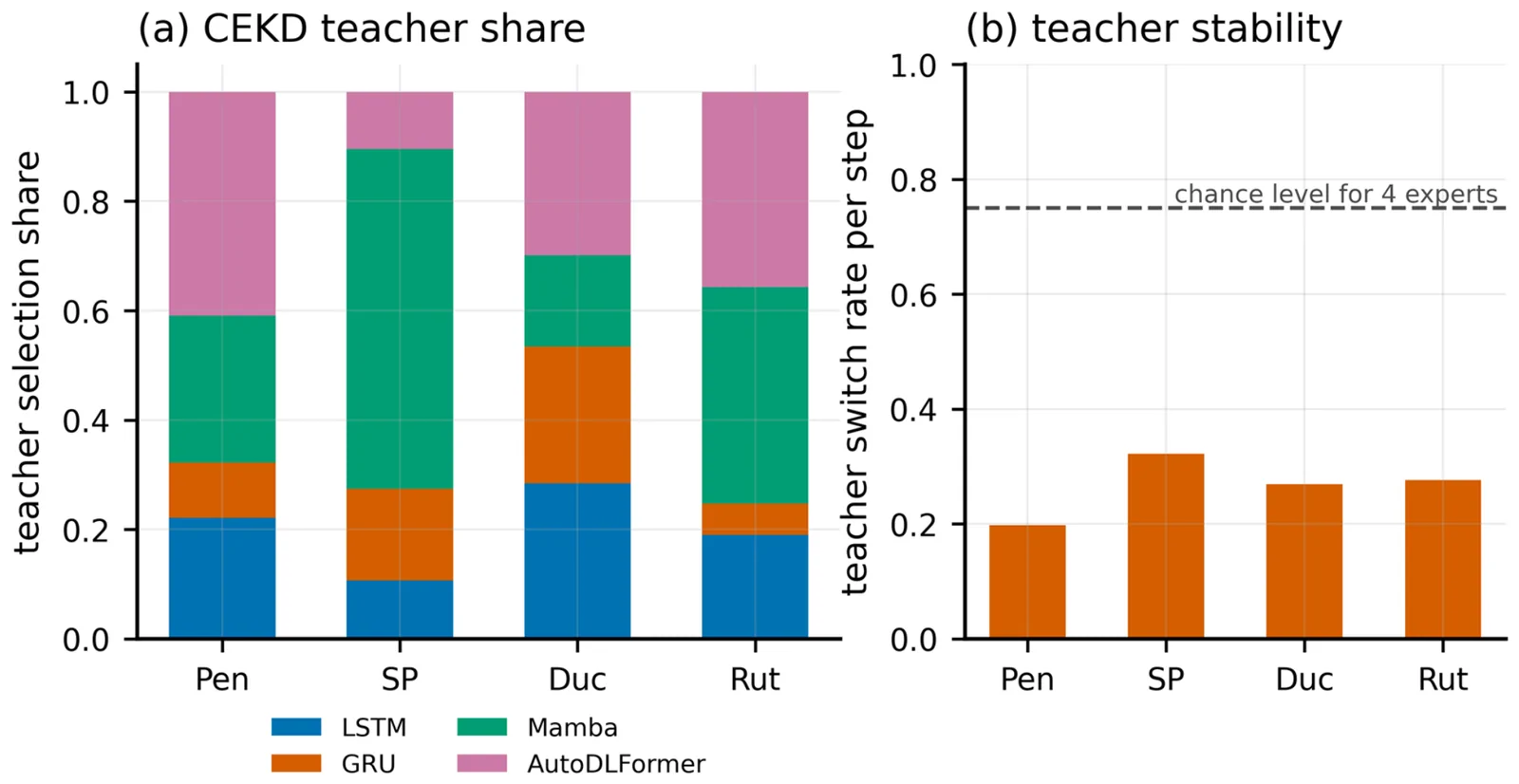
Behaviour of the cross-expert distillation term. (**a**) Fraction of optimisation steps for which each expert was selected as the teacher. (**b**) Rate at which the teacher identity changes between consecutive steps; the dashed line is the rate expected under random selection among four experts.

The distillation term behaves similarly (Figure 6): the teacher identity changes on 20%/32%/27%/28% of consecutive optimisation steps for the four tasks, and the most frequently selected expert holds between 30 and 62 percent of the steps. A teacher that changes on a large fraction of steps is not selecting a stable specialist; it is enforcing mutual consistency among experts from step to step. That may act as a regularisation mechanism, but the lambda sweep in Section 4.3 does not show a robust incremental benefit, and it is not the specialisation mechanism the term was named for.

Component evidence. Each original neural component was removed in turn, and the model was retrained under the nested protocol: removing AdaptiveGate changes mean pooled R^2^ by −0.048; removing LATN changes mean pooled R^2^ by −0.026; removing DeepPenHead changes mean pooled R^2^ by +0.008; removing Uncertainty changes mean pooled R^2^ by +0.025; removing Augmentation changes mean pooled R^2^ by +0.037; removing CEKD changes mean pooled R^2^ by +0.097. Two controls were added in response to the concern that the improvement cannot be attributed to any single component: replacing the four heterogeneous experts with four identical AutoDLFormer experts gives a mean pooled R2 of 0.286 against 0.381 for the heterogeneous model. The second control removes the input-noise augmentation. Table 7 reports all variants.

### 4.3. Robustness and Statistical Evidence (Figure 7)

Four preprocessing decisions were examined, each of which could in principle account for the reported performance.

- Skewness threshold: threshold 0.99 (as submitted) → mean R^2^ 0.381; log transform forced on all four targets → mean R^2^ 0.525; log transform disabled → mean R^2^ 0.462; threshold 0.50 → mean R^2^ 0.371; threshold 2.00 → mean R^2^ 0.399. The two degenerate rules, in which the log transform is disabled entirely or applied to all four targets, are included; the best tested rule is log transform forced on all four targets at 0.525, and the tested range spans 0.371 to 0.525. The result is materially sensitive to this preprocessing rule, so tau = 0.99 is retained only as the pre-specified main setting and not defended as optimal.
- Distillation weight: lambda = 0.00 → mean R^2^ 0.539; lambda = 0.10 → mean R^2^ 0.351; lambda = 0.30 → mean R^2^ 0.381; lambda = 0.50 → mean R^2^ 0.518; lambda = 1.00 → mean R^2^ 0.326. The auxiliary heads remain in the architecture at lambda = 0, isolating the objective from the capacity change; the best tested value is lambda = 0.00 at mean R^2^ 0.539; the lambda = 0 control is 0.539. The non-monotonic sweep and strong lambda = 0 control do not support a robust incremental CEKD benefit.
- Feature token order. Two fixed random permutations of the feature axis were applied, and five architectures were retrained under each. Mean pooled coefficient of determination, original versus permuted: TA-MoE-MTL 0.381 versus 0.361 and 0.368; LSTM 0.227 versus 0.336 and 0.382; GRU 0.436 versus 0.439 and 0.379; Transformer 0.311 versus 0.283 and 0.391; Mamba 0.416 versus 0.335 and 0.271. The proposed model changes by at most 0.020, but the largest change across the five architectures is 0.155. The sequence encoders are therefore not order-invariant, and their dependence on an arbitrary column order is a limitation rather than evidence for an unordered set representation.
- Prediction clipping. Both clipped and unclipped predictions were stored for every configuration: clipping changes pooled R^2^ by at most 0.015 on the three consistency targets and by up to 0.000 on the rutting factor.
- Rubber dosage units: as recorded, 0.300/0.052/0.695/0.478 versus unit-harmonised 0.382/0.279/0.739/0.300 (mean R^2^ 0.381 versus 0.425; largest per-task change +0.227).

**Figure 7:**
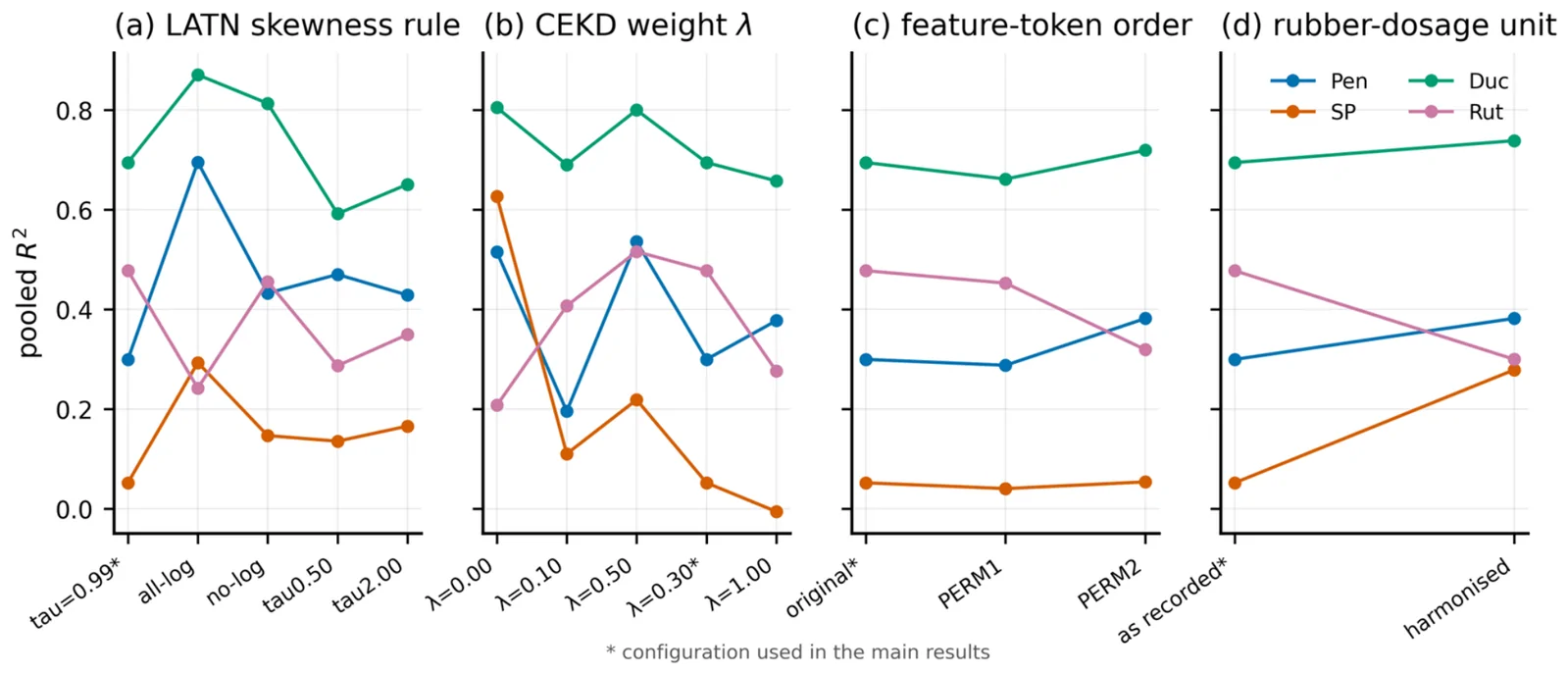
Sensitivity of the pooled coefficient of determination to four preprocessing decisions. Asterisks mark the configuration used in the main results.

Statistical comparison. With 20 independent designs and five folds, a fold-level paired test has almost no power, and a significance statement based on it is uninformative in either direction. We therefore report effect sizes as the primary quantity: for each baseline and target, the difference in the coefficient of determination together with a 95 percent confidence interval obtained by resampling mix designs. Holm-corrected *p*-values [25] are reported as secondary information, with the inferential family declared per target rather than pooled across the four targets, since the four targets test different hypotheses.

**Figure 8:**
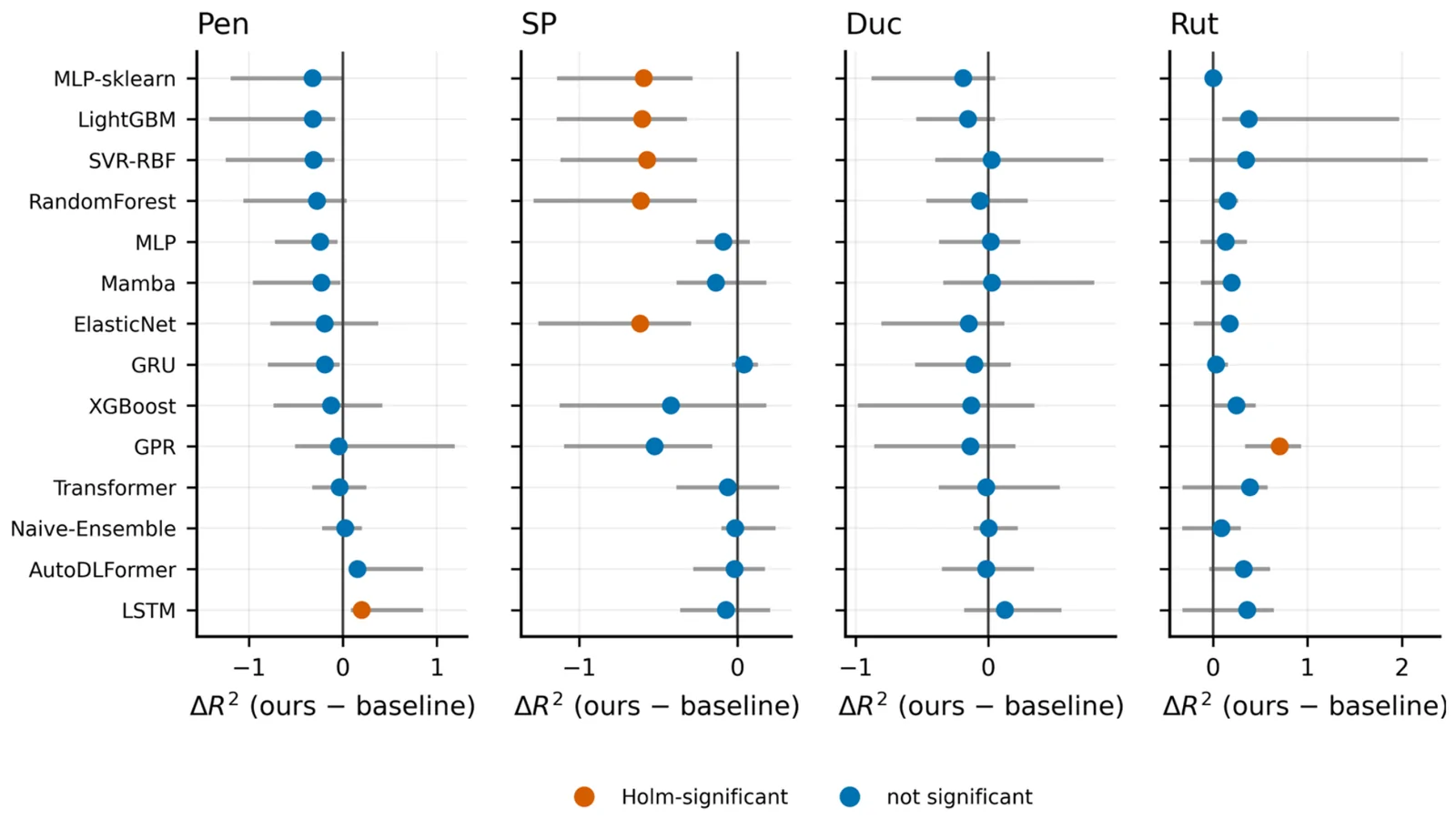
Difference in the pooled coefficient of determination between TA-MoE-MTL and each baseline, with 95 percent confidence intervals obtained by resampling mix designs. Positive values favour the proposed model.

For the original TA-MoE-MTL comparison, 56 comparisons were made; seven survive task-wise Holm correction under the recipe-level bootstrap and none under the fold-level Wilcoxon test used in the submitted version. Two significant effects favour TA-MoE-MTL (Penetration versus LSTM, Rutting Factor versus GPR), whereas five favour the baseline on Softening Point. For the locked hybrid versus MLP–sklearn (Table 8), task-wise R^2^ differences from MLP–sklearn are −0.009/−0.049/−0.006/+0.211; none of the four survives Holm correction, and all four 95% recipe bootstrap intervals include zero. More broadly, the 95 percent interval of the R^2^ difference covers zero in 40 of 56 comparisons (Figure 8). The honest summary is that this dataset cannot distinguish between most of the models evaluated on it, and that the intervals—not the *p*-values—make that visible.

**Figure 9:**
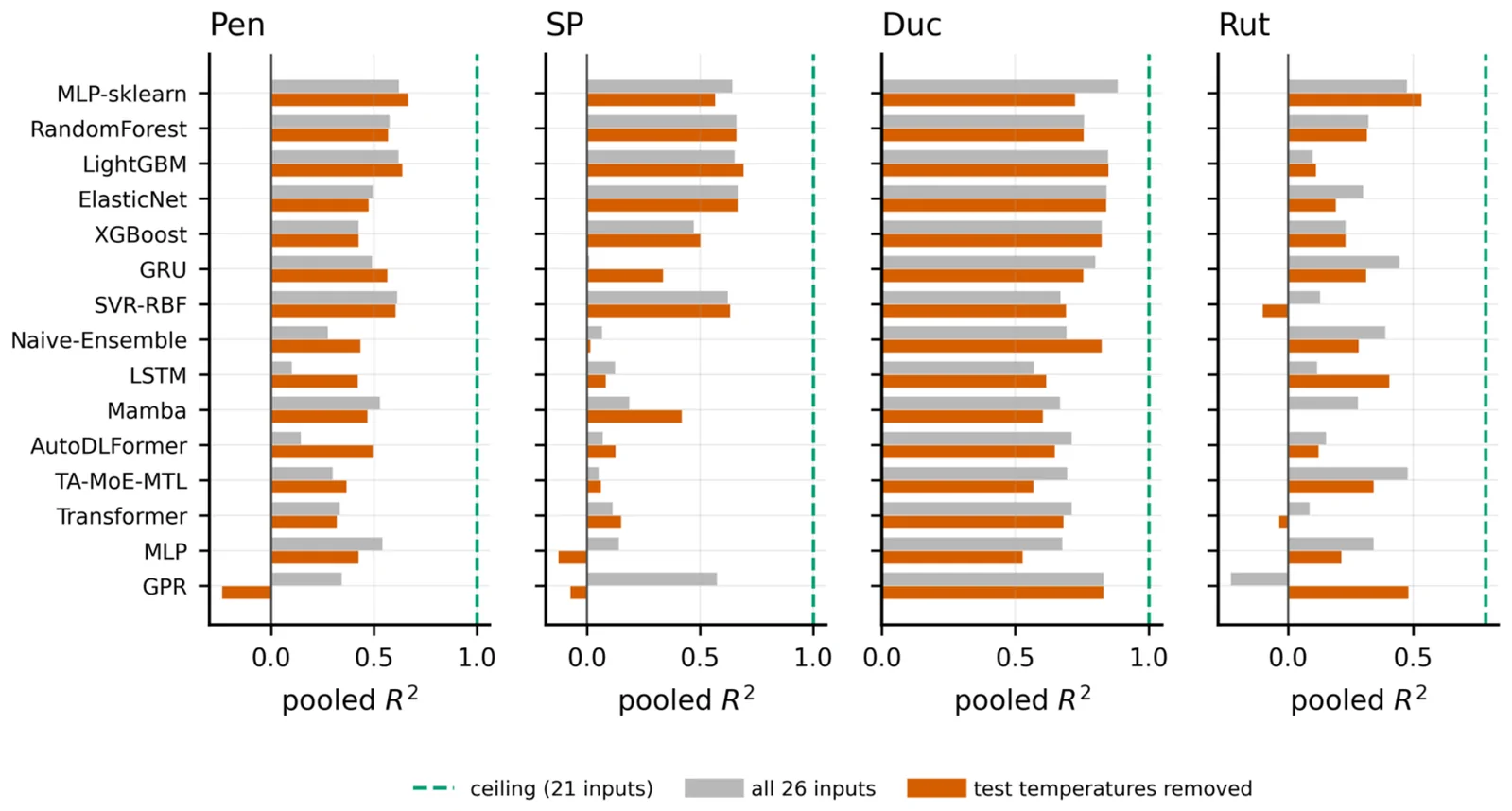
Effect of removing the five test condition inputs. All models were retrained; the dashed line is the attainable ceiling for the reduced input set.

Removal of test condition inputs. The three test condition temperatures describe the measurement rather than the material, and a model intended for mix-design screening will not have them at prediction time. All fifteen configurations were therefore retrained with the five test condition inputs removed (Figure 9). The best mean pooled R^2^ is 0.623 (MLP–sklearn), and TA-MoE-MTL reaches 0.335. For the locked final model, the locked hybrid reaches a mean pooled R2 of 0.688 without the five test condition inputs, 0.065 above the best retrained reference. Without those inputs, the design matrix collapses to 20 distinct rows, so the attainable coefficient of determination for the rutting factor is 0.790; the best rutting factor R^2^ is 0.534 (MLP–sklearn), while TA-MoE-MTL reaches 0.342. The achieved values remain well below the bound, so both missing input information and model/small-sample error limit performance in this configuration.

### 4.4. Generalization, Error Analysis and Interpretation

Interpolation and extrapolation. Holding out complete mix designs prevents replicate leakage, but it does not by itself establish that the model extrapolates to formulations unlike those it has seen. For every held-out design, we computed its nearest neighbour distance to the training designs in the standardised mix-design space, and flagged designs that fall outside the training range on any variable. Two of 20 designs are range-extrapolating in the fold in which they are held out.

**Figure 10:**
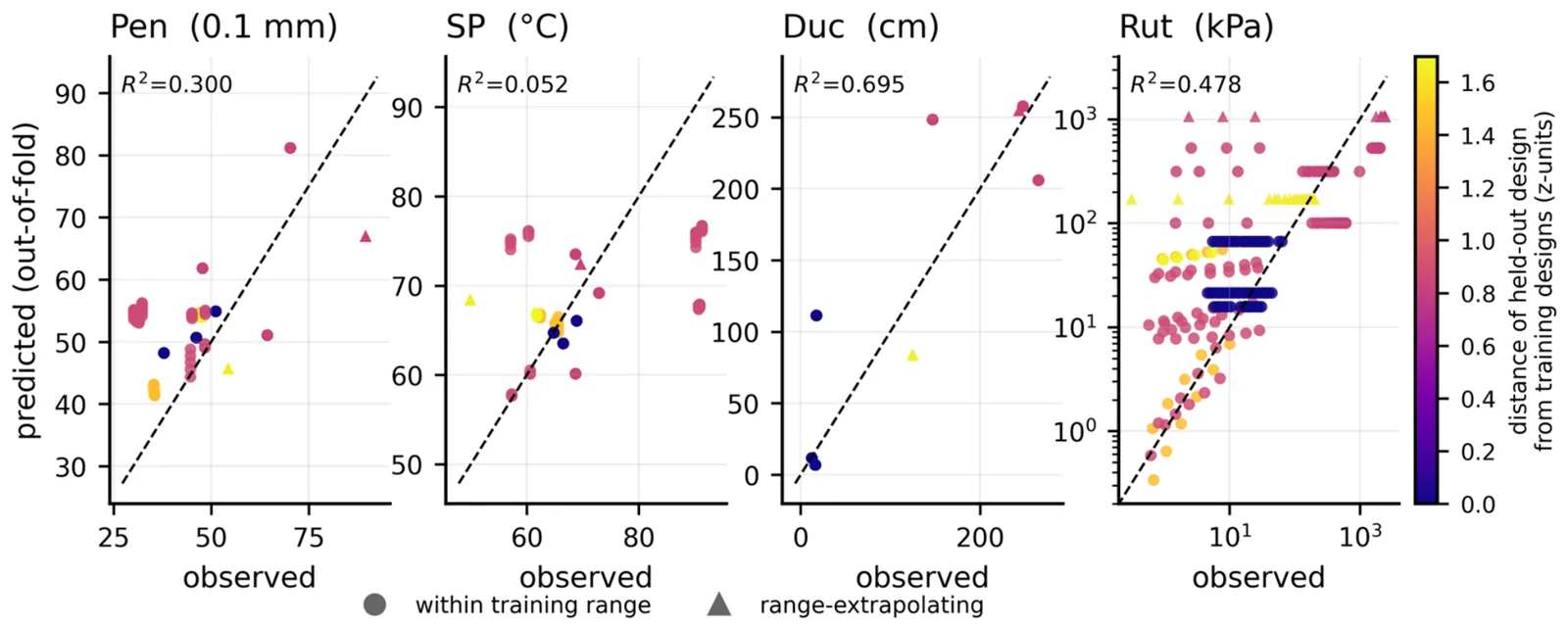
Observed versus predicted values for held-out designs, coloured by the distance of the design from the training designs in the standardised mix-design space. Triangles mark designs that fall outside the training range on at least one variable. The rutting factor panel uses logarithmic axes.

Figure 10 colours the observed-versus-predicted scatter by that distance. The recipe-level correlations between mean absolute error and distance are −0.016/0.143/0.094/−0.046 for the four tasks; there is no stable monotonic relationship in twenty designs. The distance is therefore used only to describe domain support, not as evidence that error grows predictably with extrapolation. We describe the results throughout as recipe-level interpolation within a sampled design region, and make no claim of transfer to other binder families.

**Figure 11:**
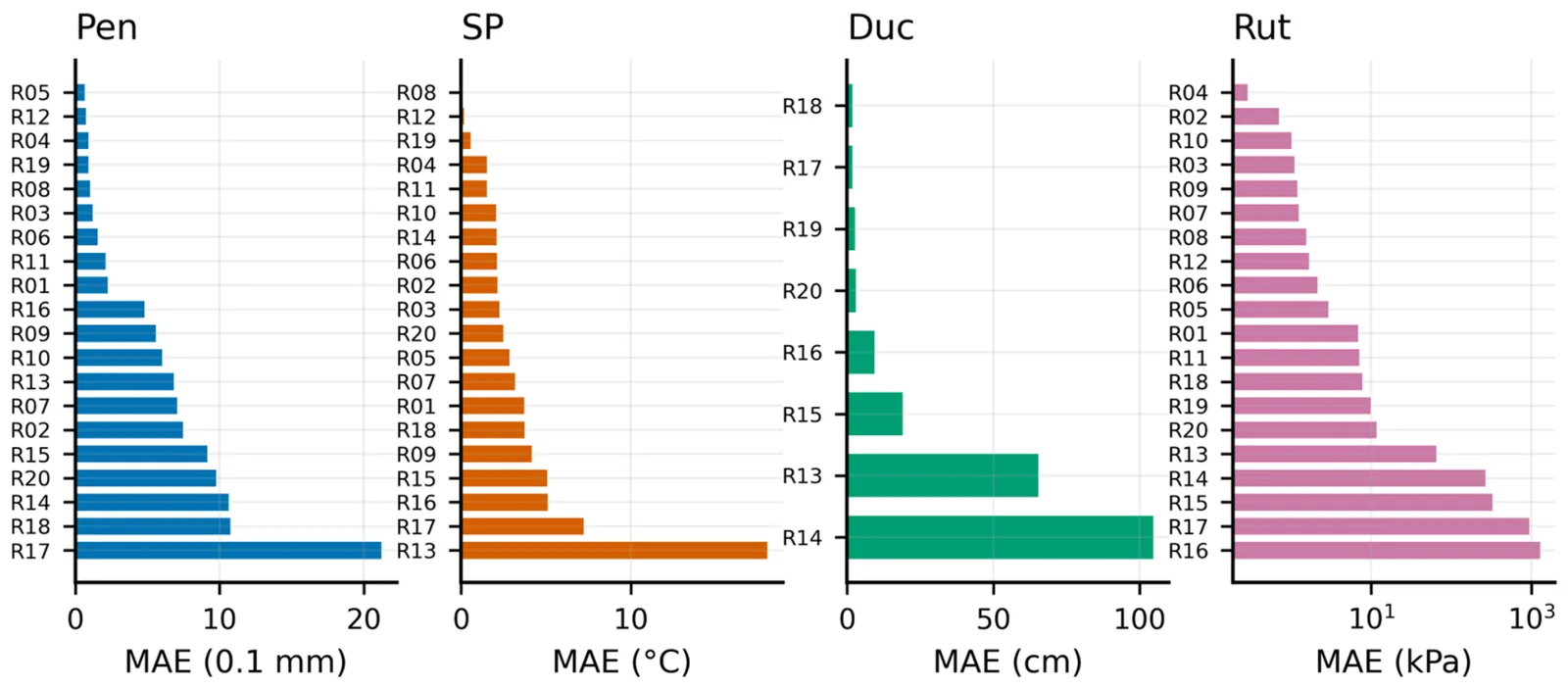
Mean absolute error per mix design under leave-one-recipe-out validation. The rutting factor panel uses a logarithmic axis.

Leave-one-recipe-out validation. Five folds over 20 designs leave only three to five designs per fold, so the estimate depends on the partition. Repeating the evaluation with one design held out at a time removes the partition entirely (Figure 11). For the original neural model, leave-one-recipe-out over all 20 designs gives pooled R^2^ values of 0.670/0.617/0.818/0.507 for penetration, softening point, ductility, and rutting factor, respectively; for the locked hybrid, the locked hybrid obtains LORO R^2^ values of 0.429/0.592/0.897/0.681 (mean 0.650), 0.003 below the best LORO reference. For ductility, where the concern is sharpest because only eight designs contribute, the leave-one-recipe-out coefficient of determination is 0.818. LORO trains on nineteen designs per fold rather than the fifteen to seventeen available in five-fold validation, so its higher pooled values cannot be attributed to partition removal alone; it is a sensitivity analysis with a larger training set per fit.

**Figure 12:**
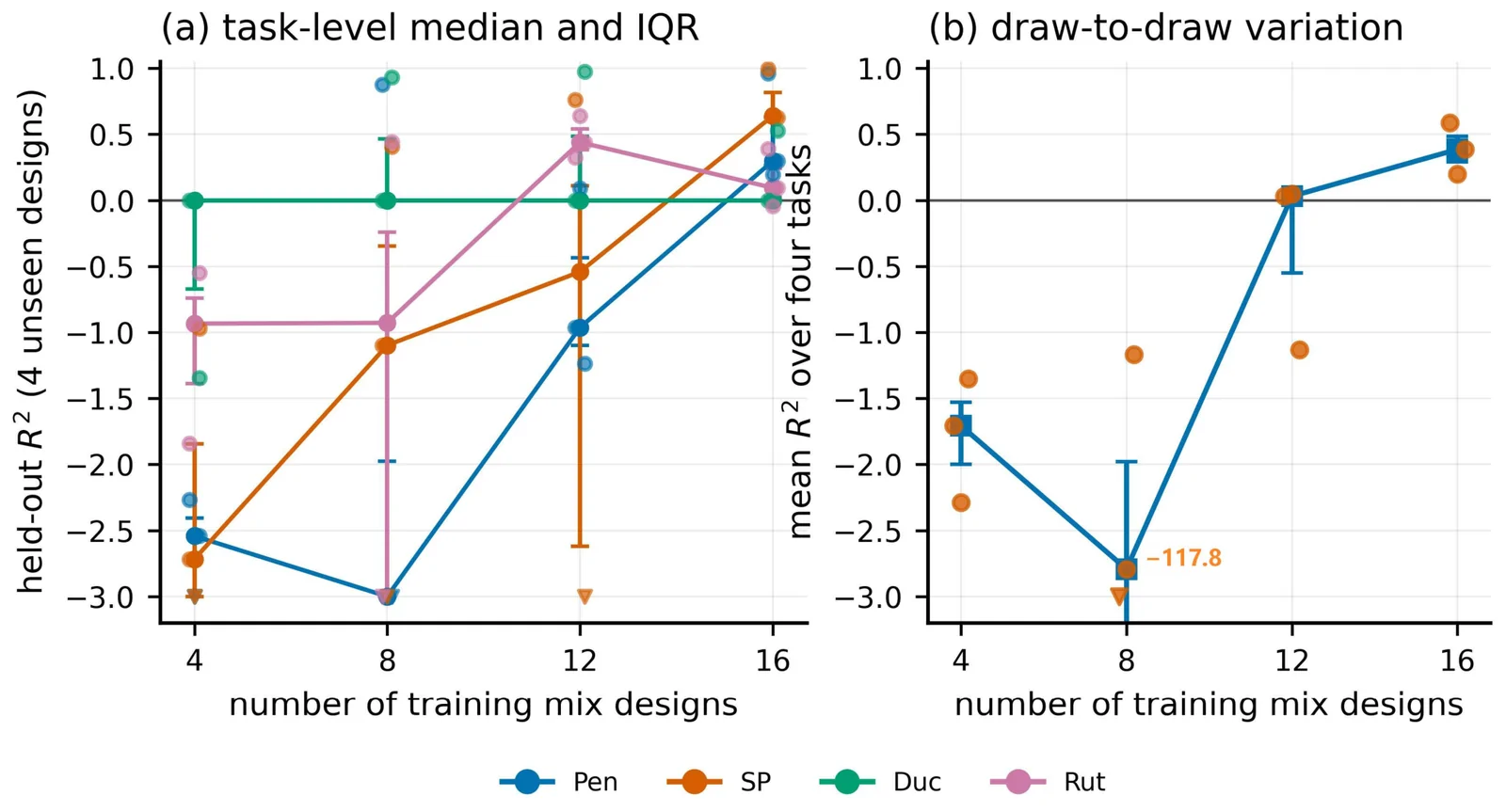
Held-out performance as a function of the number of training mix designs, over three independent draws of the training set. Lines show medians, error bars show interquartile ranges, and small points show individual draws. Downward triangles at the display floor denote values below −3; the most extreme value is labelled explicitly.

Learning curve (Figure 12). For each of three independent draws, four designs were reserved as the test set, and the model was trained on 4, 8, 12, and 16 of the remaining designs. Mean coefficient of determination over the four targets: 4 designs → −1.783 ± 0.474; 8 designs → −40.597 ± 66.892; 12 designs → −0.351 ± 0.675; 16 designs → 0.390 ± 0.194. The curve is still rising at the largest size these data allow, with a gain of +0.741 between the two largest sizes, but it is non-monotonic, and the most unstable draw occurs with eight training designs (seed 0, mean R^2^ −117.832). This is direct evidence that the model is operating in a high-variance, small-data regime and that the reported accuracy should not be read as an estimate of what the architecture could achieve with adequate data.

Attribution and its limits. Attributions are obtained by fitting a gradient-boosted surrogate to the held-out predictions of the network and computing exact tree Shapley values on the surrogate [26]. Two different questions must be separated before those values can be read, and the submitted version of this work conflated them.

The first question is whether the surrogate reproduces the network over the inputs that were actually sampled. If it does not, its attributions describe the surrogate and not the model. Under random five-fold splitting, the surrogate reproduces the network almost exactly: the coefficient of determination between surrogate and network is 0.998/0.985/1.000/1.000 for penetration, softening point, ductility, and the rutting factor, respectively. The second question is whether the surrogate still reproduces the network on mix designs that it has not seen. Under mix-design-grouped splitting, it does not, for any target: the corresponding values are −16.30/−3.92/−908.72/−9.39. Together, the two answers define exactly what may be claimed (Table 9). The attributions faithfully describe how the fitted model uses its inputs inside the sampled design region; they are not evidence about formulations outside it, and no mechanistic claim that requires extrapolation is made from them.

**Figure 13:**
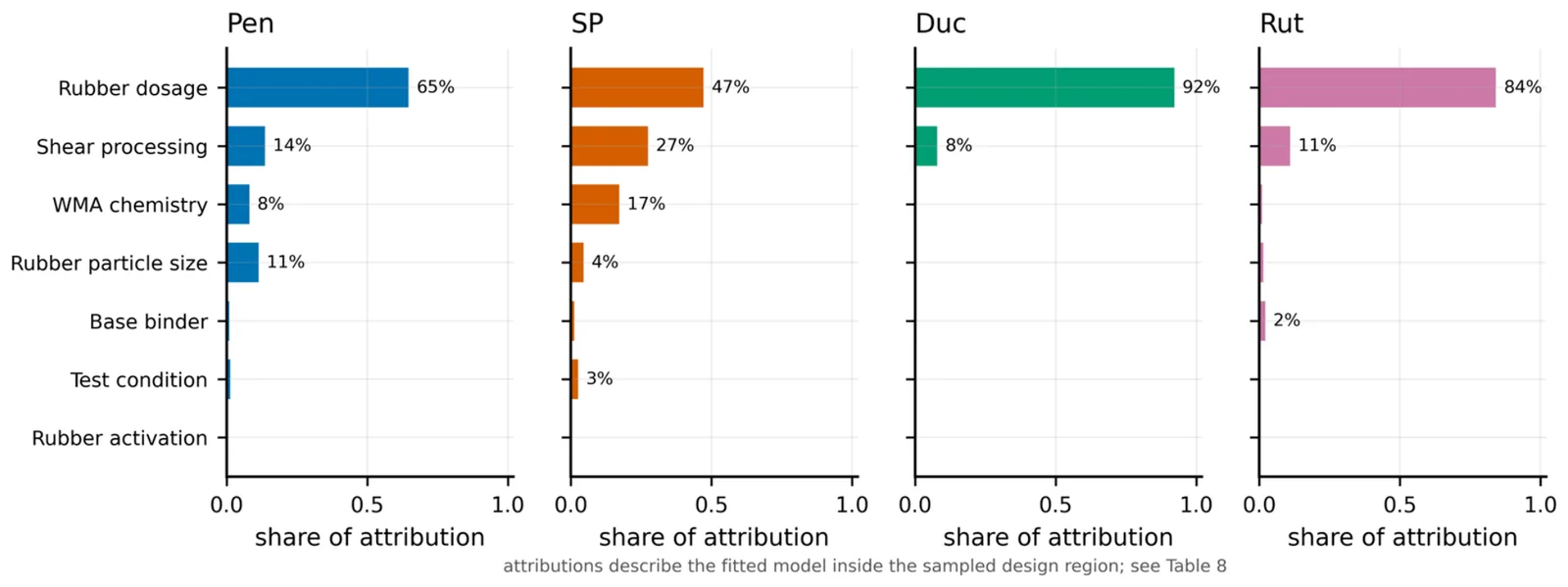
Attributions aggregated by physical factor. Bars give the summed mean absolute Shapley value of all inputs belonging to each factor, normalised within each target. These describe the fitted model inside the sampled design region only.

Within that restriction, attributions are aggregated into seven physical factors before interpretation (Figure 13), because several inputs are deterministic transforms of the same underlying quantity and column-level importance would otherwise be split arbitrarily between them. Penetration: Rubber dosage 65 percent, Shear processing 14 percent; Softening Point: Rubber dosage 47 percent, Shear processing 27 percent; Ductility: Rubber dosage 92 percent, Shear processing 8 percent; Rutting Factor: Rubber dosage 84 percent, Shear processing 11 percent. The dominance of rubber dosage is consistent with the mechanism usually invoked for CRMA, in which dosage governs swelling and the formation of an elastic gel network while shear input governs how completely that network develops. It is worth noting what the aggregation changes: read column by column, the dosage signal is divided among the raw dosage, the effective dosage transform, and the dosage-to-additive ratio, which understates it and inflates the apparent contribution of unrelated variables.

## 5. Discussion

### 5.1. What the Evidence Supports

Supported. Under mix-design-grouped five-fold cross-validation with nested, group-aware model selection, all sixteen configurations attain the performance reported in Table 6 and Table 7, and the measurement protocol behind those numbers closes both the replicate leakage path and the model selection leakage path. The optimistic bias of the more common protocol is +0.244 (range +0.024 to +0.412 over the eight headline models).

Supported. The recorded inputs bound what any deterministic model can achieve at 1.000, 1.000, 1.000, and 0.790 for the four targets, and the locked hybrid reaches 87% of that bound on the rutting factor.

Supported with a boundary. The locked hybrid ranks first of 16 models at a mean pooled R^2^ of 0.693, exceeding MLP–sklearn (0.656) by 0.037, and seeds 42/43/44 give mean pooled R^2^ values of 0.693/0.689/0.693 (mean 0.691 ± 0.002), and all three runs remain above the frozen MLP–sklearn reference. Not supported is uniform or statistically resolved task-wise superiority: task-wise R2 differences from (MLP–sklearn) are −0.009/−0.049/−0.006/+0.211; none of the four survives Holm correction, and all four 95% recipe bootstrap intervals include zero. We withdraw the broader claim made for the original model in the submitted version.

Not supported. Transfer to other binder families, other formulation regions, or other measurement campaigns. All measurements come from one CRMA family, and two of 20 held-out designs already fall outside the training range on at least one variable.

### 5.2. Does the Additional Complexity Pay for Itself?

For the neural architecture alone, no. The original network has approximately 513,000 trainable parameters against approximately 11,800 for the MLP–sklearn reference, and the learning curve in Figure 12 indicates that the training set is far from the size at which the extra capacity could be identified. It is worth being precise about what this does and does not imply. It does not imply that mixture-of-experts modelling is inappropriate for binder property prediction; it implies that 20 independent designs cannot distinguish it from a much simpler model. The locked hybrid ranks first only after the robust Huber anchor receives 90% of the prediction weight. Any study reporting a neural complexity advantage on a dataset of this size should therefore be read with the protocol in mind.

The engineering reading is also worth stating. The penetration errors correspond to roughly one penetration grade and the softening point errors to several degrees Celsius, both of which exceed the tolerances used in specification work and in the distress models that consume these properties [27]. No model evaluated here is ready to replace laboratory characterisation for mix-design screening. What the exercise does provide is a defensible measurement of how far the field is from that goal on data of this kind.

### 5.3. Relation to Accuracies Reported in the Literature

Coefficients of determination above 0.9 are common in the CRMA machine learning literature. A recent study of ground-tyre-rubber binders, for instance, reports a test-set R^2^ of 0.9571 for the complex shear modulus from 72 specimens [28], and comparable values are reported for penetration and softening point on datasets of a few dozen samples [7]. The numbers reported here are lower, and the difference is a matter of question being asked rather than of method quality. Under random splitting of the same measurements, temperature replicates of a design appear on both sides of the split, and a model is scored partly on its ability to recognise formulations it has already seen. Our own data reproduce that inflation, and the model selection effect measured in Table 5 adds to it. This is not specific to binder modelling: recent pavement management review and prediction literature emphasises that reported accuracy must be read together with the validation design that produced it [29,30]. We suggest that future studies on replicated binder datasets report the random, grouped, and nested-grouped protocols side by side, so the leakage gap is visible rather than implicit.

### 5.4. Limitations

- Effective sample size. Twenty independent mix designs, of which eight carry ductility. The learning curve is still rising at 16 training designs.
- Within-design constancy of the consistency targets. Penetration, softening point, and ductility take 20, 19, and 8 distinct values, respectively; the row count contributes no additional target information for these three.
- Identification failure for the rutting factor. Eight designs share identical input vectors while their targets differ, placing 21.0% of the variance beyond reach.
- Unit ambiguity in the rubber dosage column for three designs, reported as recorded with a sensitivity analysis rather than silently corrected.
- Single binder family and a single laboratory campaign; no external validation set exists.
- Surrogate-based attribution is high-fidelity inside the sampled region for all four tasks but fails under mix-design-grouped splitting for every task. The reported factor attributions are therefore exploratory in-domain descriptions, not evidence of mechanistic transfer to unseen formulations.
- G*/sin δ is the conventional Superpave rutting indicator but a limited proxy for modified binders; the multiple stress creep recovery parameter is the modern alternative [31] and was not measured in this campaign.
- No calibrated prediction intervals. Conformal prediction under the same group structure is the natural extension and is not attempted here.

## 6. Conclusions

(1) We define and apply an evaluation protocol for replicated mix-design datasets in which folds are grouped by mix design, all preprocessing is fitted inside the training fold, and the stopping epoch is selected on an inner group-disjoint split. Choosing the stopping epoch on the reported fold instead inflates the mean coefficient of determination by +0.244 (range +0.024 to +0.412 over the eight headline models), and the inflation is not uniform across architectures: the two protocols rank the eight headline models with a Spearman correlation of −0.02; MLP is first under the submitted protocol but second of eight under the nested one, where GRU is first, and GRU moves seven places.

(2) We bound what the recorded inputs can explain before fitting any model. For the four targets, the attainable coefficients of determination are 1.000, 1.000, 1.000, and 0.790; the rutting factor bound is below unity because eight designs share identical input vectors while their measured values differ. Reporting performance against this bound changes its interpretation: the locked hybrid reaches 87% of what the rutting factor data allow.

(3) Under this protocol, the locked hybrid ranks first of 16 models at a mean pooled R^2^ of 0.693, exceeding MLP–sklearn (0.656) by 0.037. The three-seed results are seeds 42/43/44, giving mean pooled R2 values of 0.693/0.689/0.693 (mean 0.691 ± 0.002), and all three runs remain above the frozen MLP–sklearn reference. This ranking is driven mainly by the rutting factor, and task-wise R^2^ differences from MLP–sklearn are −0.009/−0.049/−0.006/+0.211; none of the four survives Holm correction, and all four 95% recipe bootstrap intervals include zero; it is therefore an average metric result rather than evidence of uniform superiority.

(4) The component diagnostics do not support the mechanism the architecture was named for. Gate entropy is close to uniform, the distillation teacher changes frequently, and the best tested value is lambda = 0.00 at mean R^2^ 0.539; the lambda = 0 control is 0.539. The component effects are non-uniform rather than consistently beneficial, so neither task-adaptive routing nor CEKD can be identified as a robust source of improvement in these data. The locked hybrid consequently relies on the locked prediction rule, which assigns 90% to a Huber-anchored robust expert branch and 10% to a freshly trained TA-MoE-MTL branch in every outer fold.

(5) The learning curve over the number of training designs is still rising at the largest size these data allow, but it is non-monotonic, and the most unstable draw occurs with eight training designs (seed 0, mean R^2^ −117.832). Any conclusion about architecture drawn from twenty designs, including ours, should be treated as provisional.

### Recommendations for Future Data Campaigns

The most valuable change available to this line of work is not a better model but a better recorded experiment. Specifically: record the actual sweep temperature of every rheological measurement, which would remove the identification failure documented in Section 2.4 and raise the rutting factor ceiling from 0.790 towards unity; record consistency properties per replicate rather than per design, so that measurement repeatability is separable from model error; expand materially beyond 20 independent designs and continue collection until a pre-specified uncertainty or learning curve stopping criterion is met, because the present four-point curve cannot justify a numerical target; and adopt the multiple stress creep recovery parameter as the rutting target. We release the complete out-of-fold prediction set, the design inventory, and the evaluation code so that these recommendations can be checked against the same data.

## Figures and Tables

**Table 1:** Inventory of the 20 independent mix designs. Rubber and additive dosages are given as recorded; see Section 2.4 for the unit inconsistency affecting R18 to R20.

Recipe	Rows	Shear Time (min)	Shear Speed (rpm)	Rubber (wt%)	WMA (wt%)	Mesh	Activation	WMA Type	Ductility Rows
**R01**	5	60.000	8000.0	20.000	0.000	40.000	0.000	0.000	0
**R02**	4	90.000	8000.0	20.000	0.000	40.000	0.000	0.000	0
**R03**	5	60.000	8000.0	20.000	5.000	40.000	0.000	2.000	0
**R04**	4	90.000	8000.0	20.000	5.000	40.000	0.000	2.000	0
**R05**	7	60.000	8000.0	20.000	3.000	40.000	0.000	1.000	0
**R06**	7	90.000	8000.0	20.000	3.000	40.000	0.000	1.000	0
**R07**	6	60.000	8000.0	20.000	0.000	80.000	0.000	0.000	0
**R08**	4	90.000	8000.0	20.000	0.000	80.000	0.000	0.000	0
**R09**	5	60.000	8000.0	20.000	5.000	80.000	0.000	2.000	0
**R10**	5	90.000	8000.0	20.000	5.000	80.000	0.000	2.000	0
**R11**	7	60.000	8000.0	20.000	3.000	80.000	0.000	1.000	0
**R12**	7	90.000	8000.0	20.000	3.000	80.000	0.000	1.000	0
**R13**	19	15.000	3000.0	0.000	0.000	30.000	0.000	0.000	19
**R14**	19	15.000	3000.0	20.000	0.000	30.000	0.000	0.000	19
**R15**	19	15.000	3000.0	30.000	0.000	30.000	0.000	0.000	19
**R16**	19	15.000	3000.0	40.000	0.000	30.000	0.000	0.000	19
**R17**	19	15.000	3000.0	50.000	0.000	30.000	0.000	0.000	19
**R18**	14	0.000	7000.0	0.200	0.000	30.000	3.000	0.000	14
**R19**	23	0.000	7000.0	0.400	0.000	30.000	3.000	0.000	23
**R20**	23	0.000	7000.0	0.300	0.000	30.000	3.000	0.000	23

**Table 2:** Derived variables, their definitions, the mechanism each is intended to encode, and the assumption on which the encoding rests.

Derived Variable	Definition	Mechanism It Encodes	Assumption
**Shear energy**	shear time × shear speed/1000	Cumulative mechanical work delivered during blending, which controls rubber devulcanisation and dispersion	Power draw is approximately constant during blending, so energy is proportional to the time–speed product
**Rubber-to-additive ratio**	rubber dosage/(WMA dosage + 0.5)	Competition between the swelling rubber phase and the viscosity-reducing additive	The offset of 0.5 wt% keeps the ratio finite for additive-free designs and is not physically meaningful in itself
**Additive present**	1 if WMA dosage > 0, else 0	Presence of a warm mix chemistry, which changes the blending regime qualitatively rather than continuously	The qualitative effect of introducing an additive is separable from its dosage effect
**Activation present**	1 if activation method is not ‘none’, else 0	Whether the rubber surface was chemically or thermally activated before blending	All activation routes share a common first-order effect on surface reactivity
**Effective rubber content**	rubber dosage × (1 + 0.1 × activation present)	Activated rubber participates more fully in network formation than the same mass of untreated rubber	The enhancement factor of 10 percent is a fixed prior, not a fitted quantity
**Log particle size**	log(1 + particle size in mesh)	Specific surface area scales non-linearly with mesh number	Mesh number is monotonically related to the reciprocal of particle diameter over the tested range
**Base stiffness index**	base softening point/(base penetration + 1)	Consistency of the base bitumen before modification, in a single dimensionless quantity	Softening point and penetration are inversely related for unmodified bitumen
**Ductility temperature difference**	ductility test temperature − base ductility test temperature	Offset between the temperature at which the modified and the base binder were tested	Ductility differences are comparable only after accounting for the test temperature offset
**Squared DSR temperature**	(DSR test temperature)^2/100	Curvature of the rheological response with temperature	The recorded DSR temperature corresponds to the temperature actually applied; Section 2.4 shows this fails for eight designs
**Shear sufficiency**	tanh(shear time/30 min)	Saturating benefit of longer blending once the rubber is dispersed	The characteristic time of 30 min is a fixed prior taken from the blending protocol
**Activation one hot (4 columns)**	indicator of each activation route	Route-specific chemistry beyond the binary activation flag	The four routes are mutually exclusive
**Additive-type one hot (3 columns)**	indicator of each additive type	Chemistry-specific behaviour of each warm mix additive	The three additive types are mutually exclusive

**Table 3:** Information content of the recorded inputs. The attainable coefficient of determination is computed from Equation (1) over classes of identical input vectors.

Target	Measurements	Distinct Input Vectors	Distinct Target Values	Contributing Designs	Attainable R^2^	Unreachable Variance
**Penetration**	221	74	20	20	1.000	0.000
**Softening Point**	221	74	19	20	1.000	0.000
**Ductility**	155	8	8	8	1.000	0.000
**Rutting Factor**	221	74	213	20	0.790	0.210

**Table 4:** Composition of the five mix-design-grouped folds, with the number of validation measurements, the number of contributing designs, and the mean and standard deviation of each target within the fold.

Fold	Val. Rows	Val. Designs	Held-Out Designs	Duc. Rows	Mean Rutting (kPa)	SD Rutting (kPa)
**1**	46	5	R04 R05 R10 R12 R19	23	12.070	11.450
**2**	46	5	R01 R02 R06 R11 R20	23	14.070	14.200
**3**	43	3	R03 R13 R16	38	734.800	863.920
**4**	43	3	R09 R14 R17	38	1012.9	1012.6
**5**	43	4	R07 R08 R15 R18	33	127.970	192.270

**Table 5:** Effect of the model selection protocol. Values are pooled out-of-fold coefficients of determination; the last column is the difference in the mean across the four targets.

Model	Nested	Submitted	Bias
**LSTM**	0.227	0.639	0.412
**AutoDLFormer**	0.269	0.581	0.312
**TA-MoE-MTL**	0.381	0.652	0.271
**Naive-Ensemble**	0.355	0.622	0.267
**MLP**	0.425	0.677	0.252
**Transformer**	0.311	0.536	0.225
**Mamba**	0.416	0.603	0.187
**GRU**	0.436	0.460	0.024

**Table 6:** Model comparison under the nested mix-design-grouped protocol. The locked hybrid is evaluated from the same outer-fold predictions as every reference model.

Rank	Model	Pen	SP	Duc	Rut	Mean
**1**	Locked Hybrid TA-MoE-MTL	0.613	0.594	0.878	0.685	0.693
**2**	MLP–sklearn	0.622	0.643	0.884	0.475	0.656
**3**	RandomForest	0.575	0.662	0.758	0.321	0.579
**4**	ElasticNet	0.493	0.666	0.842	0.300	0.575
**5**	LightGBM	0.619	0.653	0.848	0.098	0.555
**6**	SVR-RBF	0.612	0.622	0.669	0.128	0.508
**7**	XGBoost	0.425	0.472	0.823	0.229	0.487
**8**	GRU	0.491	0.010	0.800	0.445	0.436
**9**	MLP	0.541	0.141	0.676	0.342	0.425
**10**	Mamba	0.529	0.188	0.668	0.280	0.416
**11**	TA-MoE-MTL	0.300	0.052	0.695	0.478	0.381
**12**	GPR	0.343	0.575	0.830	−0.229	0.380
**13**	Naive-Ensemble	0.275	0.066	0.692	0.388	0.355
**14**	Transformer	0.334	0.113	0.711	0.086	0.311
**15**	AutoDLFormer	0.145	0.070	0.711	0.152	0.269
**16**	LSTM	0.100	0.124	0.570	0.115	0.227

**Table 7:** Component ablation under the nested protocol. Pooled coefficients of determination and the change in the mean relative to the complete model.

Variant	Pen	SP	Duc	Rut	Mean	Change vs. Full
**HomogeneousExpert**	0.243	−0.016	0.568	0.351	0.286	−0.095
**TA-MoE-MTL**	0.300	0.052	0.695	0.478	0.381	0.000
**w-o-AdaptiveGate**	0.352	0.088	0.634	0.257	0.333	−0.048
**w-o-Augmentation**	0.436	0.035	0.824	0.377	0.418	0.037
**w-o-CEKD**	0.503	0.336	0.850	0.221	0.478	0.097
**w-o-DeepPenHead**	0.467	0.083	0.824	0.180	0.389	0.008
**w-o-LATN**	0.312	0.146	0.742	0.218	0.355	−0.026
**w-o-Uncertainty**	0.439	0.375	0.710	0.099	0.406	0.025

**Table 8:** Recipe bootstrap comparison of the locked hybrid with MLP–sklearn. Intervals resample complete mix designs; *p*-values are Holm-corrected across the four tasks.

Task	R^2^ Difference	95% CI Low	95% CI High	Holm *p*
**Penetration**	−0.009	−0.223	0.123	1.000
**Softening Point**	−0.049	−0.223	0.019	0.672
**Ductility**	−0.006	−0.096	0.046	1.000
**Rutting Factor**	0.211	−1.204	0.377	0.672

**Table 9:** Fidelity of the attribution surrogate. In-domain fidelity is measured under random splitting and determines whether attributions describe the model; fidelity on unseen designs is measured under mix-design-grouped splitting and determines whether they transfer.

Target	Fidelity, In-Domain	Fidelity, Unseen Designs	Attribution Reported	Mechanistic Transfer Supported
**Penetration**	0.998	−16.299	yes	no
**Softening Point**	0.985	−3.917	yes	no
**Ductility**	1.000	−908.721	yes	no
**Rutting Factor**	1.000	−9.389	yes	no

## Data Availability

The original contributions presented in this study are included in the article. Further inquiries can be directed to the corresponding author.

## Supplementary Materials

The following supporting information can be downloaded at: https://www.mdpi.com/article/10.3390/ma19173727/s1, Section S1: Reproducibility; Section S2: Inputs and their redundancy; Section S3: Full performance tables; Section S4: Sensitivity analyses; Section S5: Inference and per-design diagnostics; Section S6: Attribution; Table S1: Definition, intended mechanism and underlying assumption for each derived variable; Table S2: Input pairs whose absolute Pearson correlation is at least 0.85; Table S3: Pooled, unclipped and recipe-balanced coefficients of determination with root-mean-square and mean absolute errors in physical units, for every model under every protocol; Table S4: Pooled coefficient of determination with and without clipping predictions to the training-fold range; Table S5: Every sensitivity configuration: skewness threshold, distillation weight, feature-token permutation and rubber-dosage unit harmonisation; Table S6: Nearest-neighbour distance of every mix design from the training designs of the fold in which it is held out; Table S7: Mean pooled coefficient of determination across seeds 42-46 for the proposed model, MLP and the no-CEKD control; Table S8: Fold-local selection over learning rate, weight decay and exponential moving averaging for all eight deep architectures; Table S9: Complete ranking after locking the Huber-anchored Hybrid TA-MoE-MTL; Table S10: Main, no-test-condition, LORO and three-seed confirmation of the locked hybrid; Table S11: Taskwise recipe-bootstrap comparison of the locked hybrid with MLP-sklearn; Table S12: Difference in the pooled coefficient of determination between TA-MoE-MTL and each baseline; Table S13: Mean absolute error per mix design under leave-one-recipe-out validation; Table S14: Out-of-fold residual diagnostics for TA-MoE-MTL under the nested protocol; Table S15: Individual learning-curve runs; Table S16: Fidelity of the attribution surrogate under random splitting and under mix-design-grouped splitting; Table S17: Share of total attribution carried by each physical factor, per target.

## Abbreviations

Every abbreviation and symbol used in this paper is defined here and again at first use in the running text.

**Abbreviation**	**Meaning**	
CRMA	crumb–rubber modified asphalt	
WMA	warm mix asphalt additive	
DSR	dynamic shear rheometer	
MSCR	multiple stress creep recovery	
CV	cross-validation	
OOF	out of fold; a prediction made for data held out of training	
MTL	multi-task learning	
MoE	mixture of experts	
TA-MoE-MTL	task-adaptive mixture-of-experts multi-task learning, the architecture evaluated here	
LATN	log-aware target normalisation	
CEKD	cross-expert knowledge distillation	
MLP	multilayer perceptron	
GRU	gated recurrent unit	
LSTM	long short-term memory network	
SHAP	Shapley additive explanations	
RMSE	root-mean-square error	
MAE	mean absolute error	
**Symbol**	**Meaning**	
n	number of laboratory measurements (rows); n = 221	
G	number of independent mix designs; G = 20	
T	number of prediction targets; T = 4	
E	number of experts in the mixture; E = 4	
d	feature dimension after engineering; d = 26	
D	latent representation dimension shared by the experts; D = 64	
x_i	engineered input vector of measurement i, an element of R^d	
y_i	target vector of measurement i, an element of R^T	
m_i	task mask of measurement i; the t-th entry is 1 when target t was measured	
g_i	mix-design label of measurement i, an element of {1, …, G}	
z_e	representation produced by expert e	
h_j	embedding of feature token j	
u_t	expert-fused representation supplied to the head of task t	
a_t	gate weight vector for target t; its entries are non-negative and sum to one	
H_t	normalised entropy of a_t; 1 denotes uniform routing	
tau	skewness threshold above which a target is log-transformed	
mu_t, nu_t	training-fold mean and standard deviation used to normalise target t	
lambda	weight of the cross-expert distillation term	
rho_delta	Huber loss with transition point delta = 1.35	
gamma, eta	strengths of the Huber-anchor prior and simplex ridge regularisation	
e_H	one-hot vector selecting the Huber expert in the robust branch	
omega	locked neural share in the final hybrid; omega = 0.10	
s_t	learnable log-variance used for homoscedastic task weighting	
R^2^	coefficient of determination on out-of-fold predictions	
R^2^_bal	coefficient of determination with every mix design weighted equally	
R^2^_max	attainable coefficient of determination given the recorded inputs, defined in Equation (1)	
**Property**	**Unit**	**Physical meaning**
Penetration	0.1 mm	resistance to needle indentation at 25 C; a consistency measure governed by asphaltene-maltene balance and rubber swelling
Softening point	degrees Celsius	ring-and-ball temperature at which the binder loses load-bearing consistency; reports the thermal stability of the rubber-asphalt gel network
Ductility	cm	elongation at break in a tensile test; measures deformation capacity across rubber-asphalt interfaces
Rutting factor	kPa	the Superpave parameter G*/sin δ for unaged binder; quantifies elastic energy storage relative to viscous dissipation at high temperature
