# Supplementary material for: Leakage-Controlled and Information-Bounded Evaluation of Multi-Task Learning for Crumb–Rubber Modified Asphalt: What Twenty Mix Designs Can and Cannot Support
Source: Materials (Basel). 2026 Sep 1;19(17):3727. doi: 10.3390/ma19173727 (PMC13567061; doi:10.3390/ma19173727)
Supplement: Supplementary file 1 [file materials-19-03727-s001.zip › materials-4492364-supplementary.pdf]

Supplementary Material

Leakage-Controlled and Information-Bounded Evaluation of Multi-Task Learning for Crumb-Rubber-Modified Asphalt

He Huang, Yingli Gao, Bin Tian, Zhuo Yang | 24 August 2026

S1. Reproducibility

Every number in the main text is produced by the deposited code from the deposited measurement table with recorded, configuration-derived random seeds. The primary comparison uses seed 42, the original-model stability audit uses seeds 42-46, and the locked hybrid confirmation uses seeds 42-44. This section lists what is deposited; it is placed here rather than in the main text because it is an inventory rather than a scientific argument.

S1.1 Computational environment

Deep models are trained with the AdamW optimiser, an initial learning rate of 1e-3, weight decay 1e-4, cosine annealing with warm restarts, batch size 32, input Gaussian noise of standard deviation 0.015 applied to standardised inputs, gradient-norm clipping at 1.0, and a maximum of 500 epochs with an early-stopping patience of 80 epochs on the inner split. The released revision runs the tabular workloads as independent one-core CPU processes and the freshly nested TA-MoE branch on a GPU. The primary seed is 42, the stability audit uses 42-46, and fold assignment is deterministic given the mix-design labels.

S1.2 Deposited artefacts

- The measurement table and the mix-design label vector.
- Out-of-fold predictions for every model, every protocol and every sensitivity configuration, stored both clipped and unclipped.
- Per-fold selected epochs and inner-validation scores for every deep configuration.
- Gate weights and cross-expert distillation teacher indices logged at every optimisation step.
- The recipe inventory, the fold composition, the information-ceiling computation and the design-space distance table.
- The scripts that generate every figure and every table in the main text and in this supplement.

S2. Inputs and their redundancy

S2.1 Derived variable definitions

The main text gives the same definitions in condensed form; the assumption column is reproduced here in full.

Table S1. Definition, intended mechanism and underlying assumption for each derived variable.

Derived variable	Definition	Mechanism it encodes	Assumption
Shear energy	shear time x shear speed / 1000	Cumulative mechanical work delivered during blending, which controls rubber devulcanisation and dispersion	Power draw is approximately constant during blending, so energy is proportional to the time-speed product
Rubber-to-additive ratio	rubber dosage / (WMA dosage + 0.5)	Competition between the swelling rubber phase and the viscosity-reducing additive	The offset of 0.5 wt% keeps the ratio finite for additive-free designs and is not physically meaningful in itself
Additive present	1 if WMA dosage > 0, else 0	Presence of a warm-mix chemistry, which changes the blending regime qualitatively rather than continuously	The qualitative effect of introducing an additive is separable from its dosage effect
Activation present	1 if activation method is not 'none', else 0	Whether the rubber surface was chemically or thermally activated before blending	All activation routes share a common first-order effect on surface reactivity
Effective rubber content	rubber dosage x (1 + 0.1 x activation present)	Activated rubber participates more fully in network formation than the same mass of untreated rubber	The enhancement factor of 10 per cent is a fixed prior, not a fitted quantity
Log particle size	log(1 + particle size in mesh)	Specific surface area scales non-linearly with mesh number	Mesh number is monotonically related to the reciprocal of particle diameter over the tested range
Base stiffness index	base softening point / (base penetration + 1)	Consistency of the base bitumen before modification, in a single dimensionless quantity	Softening point and penetration are inversely related for unmodified bitumen
Ductility temperature difference	ductility test temperature - base ductility test temperature	Offset between the temperature at which the modified and the base binder were tested	Ductility differences are comparable only after accounting for the test temperature offset
Squared DSR temperature	(DSR test temperature)^2 / 100	Curvature of the rheological response with temperature	The recorded DSR temperature corresponds to the temperature actually applied; Section 2.4 shows this fails for eight designs
Shear sufficiency	tanh(shear time / 30 min)	Saturating benefit of longer blending once the rubber is dispersed	The characteristic time of 30 min is a fixed prior taken from the blending protocol
Activation one-hot (4 columns)	indicator of each activation route	Route-specific chemistry beyond the binary activation flag	The four routes are mutually exclusive
Additive-type one-hot (3 columns)	indicator of each additive type	Chemistry-specific behaviour of each warm-mix additive	The three additive types are mutually exclusive

S2.2 Strongly correlated input pairs

41 pairs of the 26 engineered inputs exceed this threshold, which is why attributions are aggregated by physical factor in Section 4.4 of the main text rather than read column by column.

Table S2. Input pairs whose absolute Pearson correlation is at least 0.85.

Input A	Input B	Pearson r

Input A	Input B	Pearson r
base_pen_0.1mm	base_softening_point_C	-1.000
rubber_content_wt%	effective_rubber_content	1.000
ductility_test_temp_C	activation_present	-1.000
ductility_test_temp_C	activation_method_0	1.000
ductility_test_temp_C	activation_method_3	-1.000
WMA_present	WMA_type_0	-1.000
activation_present	activation_method_0	-1.000
activation_present	activation_method_3	1.000
activation_method_0	activation_method_3	-1.000
base_softening_point_C	base_stiffness_index	0.998
base_pen_0.1mm	base_stiffness_index	-0.998
DSR_test_temp_C	DSR_test_temp_C_squared	0.998
rubber_particle_size_mesh	log_particle_size	0.996
shear_time_min	shear_energy	0.992
ductility_temp_difference	shear_sufficiency	0.991
base_ductility_test_temp_C	shear_energy	-0.967
WMA_content_wt%	WMA_present	0.960
WMA_content_wt%	WMA_type_0	-0.960
base_ductility_cm	base_stiffness_index	0.957
shear_speed_rpm	base_ductility_cm	-0.954
shear_time_min	base_ductility_test_temp_C	-0.946
base_softening_point_C	base_ductility_cm	0.939
base_pen_0.1mm	base_ductility_cm	-0.938
shear_time_min	shear_sufficiency	0.919
rubber_WMA_ratio	effective_rubber_content	0.908
rubber_content_wt%	rubber_WMA_ratio	0.908
ductility_test_temp_C	ductility_temp_difference	0.879
activation_present	ductility_temp_difference	-0.879
ductility_temp_difference	activation_method_0	0.879
ductility_temp_difference	activation_method_3	-0.879
shear_energy	shear_sufficiency	0.872
base_pen_0.1mm	ductility_test_temp_C	-0.868
base_pen_0.1mm	activation_present	0.868
base_pen_0.1mm	activation_method_0	-0.868
base_pen_0.1mm	activation_method_3	0.868
base_softening_point_C	ductility_test_temp_C	0.867
base_softening_point_C	activation_present	-0.867
base_softening_point_C	activation_method_0	0.867
base_softening_point_C	activation_method_3	-0.867
shear_time_min	ductility_temp_difference	0.864
base_ductility_test_temp_C	shear_sufficiency	-0.860

S2.3 Grouping-precision audit

The analytical recipe identifiers were recomputed from the ten mix-design variables without rounding and after rounding from two to eight decimal places. the exact values and every rounding precision from two to eight decimal places all yield 20 groups. This shows that the count is numerically stable; it does not replace the independent batch identifier absent from the workbook.

S3. Full performance tables

S3.1 All models, all protocols, all metrics

This is the full-precision version of main-text Tables 6 and 7. It replaces the thirteen-column table of the submitted version, which Reviewer 3 identified as unreadable.

Table S3. Pooled, unclipped and recipe-balanced coefficients of determination with root-mean-square and mean absolute errors in physical units, for every model under every protocol.

protocol	model	task	R2_pooled	R2_pooled_unclipped	R2_recipe_balanced	RMSE	MAE
deep, nested	AutoDLFormer	Penetration	0.145	0.145	-0.184	14.699	13.204
deep, nested	AutoDLFormer	Softening Point	0.070	0.070	0.097	9.952	7.171
deep, nested	AutoDLFormer	Ductility	0.711	0.711	0.714	55.372	45.093
deep, nested	AutoDLFormer	Rutting Factor	0.152	0.152	0.232	665.847	312.330
deep, nested	GRU	Penetration	0.491	0.491	0.338	11.343	9.355
deep, nested	GRU	Softening Point	0.010	0.010	0.062	10.265	7.280
deep, nested	GRU	Ductility	0.799	0.799	0.799	46.091	38.098
deep, nested	GRU	Rutting Factor	0.445	0.445	0.498	538.807	266.606
deep, nested	LSTM	Penetration	0.100	0.100	-0.219	15.083	13.068
deep, nested	LSTM	Softening Point	0.124	0.124	0.074	9.655	6.858
deep, nested	LSTM	Ductility	0.570	0.570	0.593	67.531	59.391
deep, nested	LSTM	Rutting Factor	0.115	0.115	0.199	680.071	309.857
deep, nested	MLP	Penetration	0.541	0.541	0.385	10.765	9.140
deep, nested	MLP	Softening Point	0.141	0.141	0.195	9.564	7.174
deep, nested	MLP	Ductility	0.676	0.676	0.704	58.624	43.792
deep, nested	MLP	Rutting Factor	0.342	0.342	0.406	586.364	287.744
deep, nested	Transformer	Penetration	0.334	0.334	0.126	12.969	10.356
deep, nested	Transformer	Softening Point	0.113	0.113	0.056	9.719	6.876
deep, nested	Transformer	Ductility	0.711	0.711	0.705	55.369	39.575
deep, nested	Transformer	Rutting Factor	0.086	0.086	0.174	691.247	310.918
deep, nested	HomogeneousExpert	Penetration	0.243	0.243	0.063	13.833	12.069
deep, nested	HomogeneousExpert	Softening Point	-0.016	-0.016	0.078	10.402	7.691

protocol	model	task	R2_pooled	R2_pooled_unclipped	R2_recipe_balanced	RMSE	MAE
deep, nested	HomogeneousExpert	Ductility	0.568	0.568	0.595	67.692	56.912
deep, nested	HomogeneousExpert	Rutting Factor	0.351	0.351	0.413	582.597	281.828
deep, nested	Mamba	Penetration	0.529	0.529	0.538	10.914	9.420
deep, nested	Mamba	Softening Point	0.188	0.188	0.286	9.300	7.042
deep, nested	Mamba	Ductility	0.668	0.656	0.661	59.347	43.637
deep, nested	Mamba	Rutting Factor	0.280	0.280	0.347	613.664	291.815
deep, nested	Naive-Ensemble	Penetration	0.275	0.275	0.130	13.532	11.371
deep, nested	Naive-Ensemble	Softening Point	0.066	0.066	0.074	9.972	7.696
deep, nested	Naive-Ensemble	Ductility	0.692	0.692	0.702	57.171	47.413
deep, nested	Naive-Ensemble	Rutting Factor	0.388	0.388	0.445	565.784	270.731
deep, nested	TA-MoE-MTL	Penetration	0.299	0.299	0.107	13.303	11.371
deep, nested	TA-MoE-MTL	Softening Point	0.052	0.052	0.093	10.047	7.112
deep, nested	TA-MoE-MTL	Ductility	0.695	0.695	0.710	56.886	42.692
deep, nested	TA-MoE-MTL	Rutting Factor	0.478	0.478	0.528	522.534	259.884
deep, nested	w-o-AdaptiveGate	Penetration	0.351	0.351	0.120	12.800	10.711
deep, nested	w-o-AdaptiveGate	Softening Point	0.088	0.088	0.077	9.853	7.328
deep, nested	w-o-AdaptiveGate	Ductility	0.634	0.634	0.649	62.290	55.379
deep, nested	w-o-AdaptiveGate	Rutting Factor	0.257	0.257	0.327	623.395	313.434
deep, nested	w-o-Augmentation	Penetration	0.436	0.436	0.179	11.936	9.564
deep, nested	w-o-Augmentation	Softening Point	0.035	0.035	0.173	10.136	7.971
deep, nested	w-o-Augmentation	Ductility	0.824	0.822	0.820	43.246	28.664
deep, nested	w-o-Augmentation	Rutting Factor	0.377	0.377	0.437	570.620	259.931
deep, nested	w-o-CEKD	Penetration	0.503	0.503	0.485	11.202	9.422
deep, nested	w-o-CEKD	Softening Point	0.336	0.336	0.476	8.407	5.832
deep, nested	w-o-CEKD	Ductility	0.850	0.850	0.847	39.818	24.021
deep, nested	w-o-CEKD	Rutting Factor	0.221	0.221	0.296	638.232	285.587

protocol	model	task	R2_pooled	R2_pooled_unclipped	R2_recipe_balanced	RMSE	MAE
deep, nested	w-o-DeepPenHead	Penetration	0.467	0.467	0.320	11.603	9.568
deep, nested	w-o-DeepPenHead	Softening Point	0.083	0.083	0.153	9.882	7.610
deep, nested	w-o-DeepPenHead	Ductility	0.824	0.810	0.834	43.137	30.277
deep, nested	w-o-DeepPenHead	Rutting Factor	0.180	0.180	0.259	654.717	316.229
deep, nested	w-o-LATN	Penetration	0.312	0.312	0.130	13.185	11.041
deep, nested	w-o-LATN	Softening Point	0.146	0.146	0.193	9.533	7.271
deep, nested	w-o-LATN	Ductility	0.743	0.743	0.748	52.242	43.909
deep, nested	w-o-LATN	Rutting Factor	0.218	0.218	0.293	639.379	297.447
deep, nested	w-o-Uncertainty	Penetration	0.440	0.440	0.400	11.900	9.374
deep, nested	w-o-Uncertainty	Softening Point	0.375	0.375	0.488	8.155	5.187
deep, nested	w-o-Uncertainty	Ductility	0.710	0.710	0.724	55.399	43.131
deep, nested	w-o-Uncertainty	Rutting Factor	0.100	0.100	0.187	686.110	311.586
deep, submitted protocol	AutoDLFormer	Penetration	0.588	0.588	0.492	10.203	7.849
deep, submitted protocol	AutoDLFormer	Softening Point	0.504	0.504	0.592	7.264	4.554
deep, submitted protocol	AutoDLFormer	Ductility	0.830	0.830	0.827	42.403	30.686
deep, submitted protocol	AutoDLFormer	Rutting Factor	0.402	0.402	0.459	559.178	271.507
deep, submitted protocol	GRU	Penetration	0.300	0.300	0.371	13.295	7.988
deep, submitted protocol	GRU	Softening Point	0.669	0.669	0.799	5.937	3.168
deep, submitted protocol	GRU	Ductility	0.893	0.893	0.891	33.684	23.496
deep, submitted protocol	GRU	Rutting Factor	-0.021	-0.021	0.078	730.463	321.437
deep, submitted protocol	LSTM	Penetration	0.589	0.589	0.600	10.196	8.110
deep, submitted protocol	LSTM	Softening Point	0.716	0.716	0.859	5.503	3.301

protocol	model	task	R2_pooled	R2_pooled_unclipped	R2_recipe_balanced	RMSE	MAE
deep, submitted protocol	LSTM	Ductility	0.747	0.747	0.741	51.821	34.977
deep, submitted protocol	LSTM	Rutting Factor	0.506	0.506	0.554	507.932	246.892
deep, submitted protocol	MLP	Penetration	0.737	0.737	0.753	8.148	6.062
deep, submitted protocol	MLP	Softening Point	0.663	0.663	0.845	5.990	3.288
deep, submitted protocol	MLP	Ductility	0.878	0.878	0.876	35.891	25.722
deep, submitted protocol	MLP	Rutting Factor	0.431	0.431	0.486	545.464	258.538
deep, submitted protocol	Transformer	Penetration	0.572	0.572	0.571	10.395	7.854
deep, submitted protocol	Transformer	Softening Point	0.703	0.703	0.846	5.623	3.383
deep, submitted protocol	Transformer	Ductility	0.751	0.751	0.745	51.356	37.605
deep, submitted protocol	Transformer	Rutting Factor	0.118	0.118	0.203	679.024	304.803
deep, submitted protocol	Mamba	Penetration	0.661	0.661	0.690	9.250	7.006
deep, submitted protocol	Mamba	Softening Point	0.650	0.650	0.779	6.107	3.931
deep, submitted protocol	Mamba	Ductility	0.721	0.721	0.715	54.383	40.223
deep, submitted protocol	Mamba	Rutting Factor	0.381	0.381	0.440	569.058	272.930
deep, submitted protocol	Naive-Ensemble	Penetration	0.660	0.660	0.655	9.268	6.950
deep, submitted protocol	Naive-Ensemble	Softening Point	0.697	0.697	0.851	5.683	3.139
deep, submitted protocol	Naive-Ensemble	Ductility	0.816	0.816	0.812	44.178	30.100
deep, submitted protocol	Naive-Ensemble	Rutting Factor	0.317	0.317	0.383	597.750	290.750

protocol	model	task	R2_pooled	R2_pooled_unclipped	R2_recipe_balanced	RMSE	MAE
deep, submitted protocol	TA-MoE-MTL	Penetration	0.652	0.652	0.698	9.373	7.545
deep, submitted protocol	TA-MoE-MTL	Softening Point	0.649	0.649	0.794	6.116	3.997
deep, submitted protocol	TA-MoE-MTL	Ductility	0.802	0.802	0.797	45.839	28.257
deep, submitted protocol	TA-MoE-MTL	Rutting Factor	0.504	0.504	0.552	509.190	250.091
tabular	ElasticNet	Penetration	0.493	0.493	0.405	11.313	9.018
tabular	ElasticNet	Softening Point	0.666	0.666	0.819	5.963	3.791
tabular	ElasticNet	Ductility	0.842	0.842	0.843	40.898	34.167
tabular	ElasticNet	Rutting Factor	0.300	0.300	0.368	604.755	280.102
tabular	RandomForest	Penetration	0.575	0.575	0.636	10.358	8.260
tabular	RandomForest	Softening Point	0.662	0.662	0.839	6.001	3.495
tabular	RandomForest	Ductility	0.758	0.758	0.752	50.686	28.140
tabular	RandomForest	Rutting Factor	0.321	0.321	0.387	595.773	281.383
tabular	SVR-RBF	Penetration	0.612	0.612	0.651	9.897	8.380
tabular	SVR-RBF	Softening Point	0.622	0.622	0.818	6.342	4.251
tabular	SVR-RBF	Ductility	0.669	0.400	0.662	59.186	45.086
tabular	SVR-RBF	Rutting Factor	0.128	0.128	0.212	675.240	316.832
tabular	GPR	Penetration	0.343	0.343	0.286	12.880	10.609
tabular	GPR	Softening Point	0.575	0.575	0.794	6.730	4.699
tabular	GPR	Ductility	0.830	0.830	0.826	42.424	33.667
tabular	GPR	Rutting Factor	-0.229	-0.229	-0.111	801.524	367.836
tabular	XGBoost	Penetration	0.425	0.425	0.521	12.050	10.065
tabular	XGBoost	Softening Point	0.472	0.472	0.740	7.498	4.355
tabular	XGBoost	Ductility	0.824	0.824	0.820	43.251	23.893
tabular	XGBoost	Rutting Factor	0.229	0.229	0.304	634.903	298.384
tabular	LightGBM	Penetration	0.619	0.619	0.620	9.811	7.924
tabular	LightGBM	Softening Point	0.653	0.653	0.824	6.079	4.022
tabular	LightGBM	Ductility	0.848	0.848	0.845	40.116	21.104
tabular	LightGBM	Rutting Factor	0.098	0.098	0.186	686.502	335.678
tabular	MLP-sklearn	Penetration	0.622	0.622	0.704	9.774	7.655
tabular	MLP-sklearn	Softening Point	0.643	0.643	0.831	6.164	4.113
tabular	MLP-sklearn	Ductility	0.884	0.884	0.882	35.022	23.558

protocol	model	task	R2_pooled	R2_pooled_unclipped	R2_recipe_balanced	RMSE	MAE
tabular	MLP-sklearn	Rutting Factor	0.475	0.475	0.526	524.032	254.721

The remaining 60 rows are in the released file S_full_metrics.csv.

S3.2 Effect of prediction clipping

Clipping was raised by Reviewer 2 as a possible source of the reported accuracy. clipping changes pooled R2 by at most 0.015 on the three consistency targets and by up to 0.000 on the rutting factor

Table S4. Pooled coefficient of determination with and without clipping predictions to the training-fold range.

model	task	R2_clipped	R2_unclipped	change
AutoDLFormer	Pen	0.145	0.145	0.000
AutoDLFormer	SP	0.070	0.070	0.000
AutoDLFormer	Duc	0.711	0.711	0.000
AutoDLFormer	Rut	0.152	0.152	0.000
GRU	Pen	0.491	0.491	0.000
GRU	SP	0.010	0.010	0.000
GRU	Duc	0.800	0.800	0.000
GRU	Rut	0.445	0.445	0.000
LSTM	Pen	0.100	0.100	0.000
LSTM	SP	0.124	0.124	0.000
LSTM	Duc	0.570	0.570	0.000
LSTM	Rut	0.115	0.115	0.000
MLP	Pen	0.541	0.541	0.000
MLP	SP	0.141	0.141	0.000
MLP	Duc	0.676	0.676	0.000
MLP	Rut	0.342	0.342	0.000
Transformer	Pen	0.334	0.334	0.000
Transformer	SP	0.113	0.113	0.000
Transformer	Duc	0.711	0.711	0.000
Transformer	Rut	0.086	0.086	0.000
HomogeneousExpert	Pen	0.243	0.243	0.000
HomogeneousExpert	SP	-0.016	-0.016	0.000
HomogeneousExpert	Duc	0.568	0.568	0.000
HomogeneousExpert	Rut	0.351	0.351	0.000
Mamba	Pen	0.529	0.529	0.000
Mamba	SP	0.188	0.188	0.000
Mamba	Duc	0.668	0.657	0.011
Mamba	Rut	0.280	0.280	0.000
Naive-Ensemble	Pen	0.275	0.275	0.000
Naive-Ensemble	SP	0.066	0.066	0.000
Naive-Ensemble	Duc	0.692	0.692	0.000
Naive-Ensemble	Rut	0.388	0.388	0.000
TA-MoE-MTL	Pen	0.300	0.300	0.000
TA-MoE-MTL	SP	0.052	0.052	0.000
TA-MoE-MTL	Duc	0.695	0.695	0.000
TA-MoE-MTL	Rut	0.478	0.478	0.000
w-o-AdaptiveGate	Pen	0.352	0.352	0.000
w-o-AdaptiveGate	SP	0.088	0.088	0.000
w-o-AdaptiveGate	Duc	0.634	0.634	0.000
w-o-AdaptiveGate	Rut	0.257	0.257	0.000

model	task	R2_clipped	R2_unclipped	change
w-o-Augmentation	Pen	0.436	0.436	0.000
w-o-Augmentation	SP	0.035	0.035	0.000
w-o-Augmentation	Duc	0.824	0.822	0.002
w-o-Augmentation	Rut	0.377	0.377	0.000
w-o-CEKD	Pen	0.503	0.503	0.000
w-o-CEKD	SP	0.336	0.336	0.000
w-o-CEKD	Duc	0.850	0.850	0.000
w-o-CEKD	Rut	0.221	0.221	0.000
w-o-DeepPenHead	Pen	0.467	0.467	0.000
w-o-DeepPenHead	SP	0.083	0.083	0.000
w-o-DeepPenHead	Duc	0.824	0.810	0.015
w-o-DeepPenHead	Rut	0.180	0.180	0.000
w-o-LATN	Pen	0.312	0.312	0.000
w-o-LATN	SP	0.146	0.146	0.000
w-o-LATN	Duc	0.742	0.742	0.000
w-o-LATN	Rut	0.218	0.218	0.000
w-o-Uncertainty	Pen	0.439	0.439	0.000
w-o-Uncertainty	SP	0.375	0.375	0.000
w-o-Uncertainty	Duc	0.710	0.710	0.000
w-o-Uncertainty	Rut	0.099	0.099	0.000

S4. Sensitivity analyses

S4.1 All sensitivity configurations

All configurations run under the nested protocol with the same folds and the same seed as the main results, so the values are directly comparable with Table 6 of the main text.

Table S5. Every sensitivity configuration: skewness threshold, distillation weight, feature-token permutation and rubber-dosage unit harmonisation.

configuration	variant	Pen	SP	Duc	Rut	mean	runtime_s
CEKD_lam0.00	CEKD	0.515	0.627	0.805	0.207	0.539	5636.2
CEKD_lam0.10	CEKD	0.195	0.110	0.690	0.407	0.351	4807.2
CEKD_lam0.50	CEKD	0.536	0.219	0.800	0.516	0.518	5594.9
CEKD_lam1.00	CEKD	0.377	-0.006	0.658	0.276	0.326	5637.4
LATN_all-log	LATN	0.696	0.293	0.871	0.242	0.525	5025.9
LATN_no-log	LATN	0.433	0.147	0.813	0.456	0.462	6100.3
LATN_tau0.50	LATN	0.470	0.135	0.592	0.287	0.371	4750.7
LATN_tau2.00	LATN	0.429	0.166	0.651	0.349	0.399	5624.5
PERM1_GRU	feature_permutation	0.549	0.102	0.781	0.326	0.439	531.600
PERM1_LSTM	feature_permutation	0.410	-0.151	0.738	0.347	0.336	273.800
PERM1_Mamba	feature_permutation	0.455	0.084	0.645	0.155	0.335	3196.4
PERM1_TA-MoE-MTL	feature_permutation	0.288	0.040	0.662	0.453	0.361	5689.9
PERM1_Transformer	feature_permutation	0.345	0.114	0.502	0.170	0.283	235.000
PERM2_GRU	feature_permutation	0.343	0.098	0.735	0.342	0.379	498.400
PERM2_LSTM	feature_permutation	0.446	0.047	0.733	0.301	0.382	279.900
PERM2_Mamba	feature_permutation	0.192	0.202	0.613	0.079	0.271	5727.8
PERM2_TA-MoE-MTL	feature_permutation	0.382	0.054	0.719	0.319	0.368	4409.5
PERM2_Transformer	feature_permutation	0.517	0.124	0.730	0.192	0.391	256.100
UNITFIX_RandomForest	unit_harmonised	0.378	0.683	0.740	0.412	0.553	80.500
UNITFIX_TA-MoE-MTL	unit_harmonised	0.382	0.279	0.739	0.300	0.425	4949.7
UNITFIX_XGBoost	unit_harmonised	0.399	0.695	0.840	0.387	0.580	47.700

S4.2 Design-space distance of each held-out design

2 of 20 designs are range-extrapolating. This table underpins Figure 10 of the main text and the distinction between interpolation and extrapolation drawn in Section 4.4.

Table S6. Nearest-neighbour distance of every mix design from the training designs of the fold in which it is held out, in standardised units, with a flag for designs that fall outside the training range on at least one variable.

Recipe	nn_distance_to_training_design	range_extrapolating
R01	1.457	False
R02	1.697	False
R03	0.870	False
R04	0.870	False
R05	0.870	False
R06	0.870	False
R07	1.457	False
R08	1.457	False
R09	0.870	False
R10	0.870	False
R11	0.870	False
R12	0.870	False

Recipe	nn_distance_to_training_design	range_extrapolating
R13	1.648	True
R14	0.824	False
R15	0.824	False
R16	0.824	False
R17	0.824	True
R18	0.008	False
R19	0.008	False
R20	0.008	False

S4.3 Five-seed stability

TA-MoE-MTL 0.351 ± 0.037 (range 0.309-0.384); MLP 0.287 ± 0.086 (range 0.219-0.425); w-o-CEKD 0.375 ± 0.098 (range 0.258-0.478)

Table S7. Mean pooled coefficient of determination across seeds 42-46 for the proposed model, MLP and the no-CEKD control.

model	n_seeds	mean_R2	sd_R2	min_R2	max_R2	five_seed_ensemble_R2
w-o-CEKD	5	0.375	0.098	0.258	0.478	0.441
TA-MoE-MTL	5	0.350	0.037	0.309	0.384	0.382
MLP	5	0.287	0.086	0.219	0.425	0.328

S4.4 Nested deep-model hyper-parameter control

TA-MoE-MTL reaches mean pooled R2 0.279 and ranks 5 of 8, while GRU is first at 0.375; the selected TA-MoE-MTL setting changes across folds and its inner score is negative in 2 of 5 folds

Table S8. Fold-local selection over learning rate, weight decay and exponential moving averaging for all eight deep architectures.

model	Pen	SP	Duc	Rut	mean_R2	recipe_balanced_R2	negative_inner_folds	distinct_settings	modal_setting
GRU	0.431	0.058	0.659	0.353	0.375	0.369	2	1	lr1e-03_wd1e-04_ema0.000
MLP	0.357	-0.089	0.447	0.500	0.304	0.274	2	2	lr1e-03_wd1e-04_ema0.000
LSTM	0.237	0.068	0.421	0.420	0.287	0.251	2	4	lr1e-03_wd1e-04_ema0.990
TA-MoE-MTL	0.377	0.080	0.451	0.207	0.279	0.273	2	3	lr1e-03_wd1e-04_ema0.000
AutoDLFormer	0.323	0.076	0.613	0.103	0.279	0.286	2	3	lr3e-04_wd1e-03_ema0.000
Mamba	0.307	0.037	0.496	0.166	0.252	0.233	2	3	lr1e-03_wd1e-04_ema0.000
Naive-Ensemble	0.336	-0.005	0.313	0.293	0.235	0.210	2	3	lr3e-04_wd1e-03_ema0.990
Transformer	0.256	0.040	0.454	0.061	0.203	0.193	2	2	lr3e-04_wd1e-03_ema0.990

S4.5 Locked hybrid ranking

ranks first of 16 models at mean pooled R2 0.693, exceeding MLP-sklearn (0.656) by 0.037. the locked prediction rule assigns 90% to a Huber-anchored robust expert branch and 10% to a freshly trained TA-MoE-MTL branch in every outer fold.

Table S9. Complete ranking after locking the Huber-anchored Hybrid TA-MoE-MTL.

rank	model	R2_Penetration	R2_Softening Point	R2_Ductility	R2_Rutting Factor	mean_R2
1	Huber-anchored Hybrid TA-MoE-MTL	0.613	0.594	0.878	0.685	0.693
2	MLP-sklearn	0.622	0.643	0.884	0.475	0.656
3	RandomForest	0.575	0.662	0.758	0.321	0.579
4	ElasticNet	0.493	0.666	0.842	0.300	0.576
5	LightGBM	0.619	0.653	0.848	0.098	0.555
6	SVR-RBF	0.612	0.622	0.669	0.128	0.508
7	XGBoost	0.425	0.472	0.824	0.229	0.487
8	GRU	0.491	0.010	0.799	0.445	0.436
9	MLP	0.541	0.141	0.676	0.342	0.425
10	Mamba	0.529	0.188	0.668	0.280	0.416
11	TA-MoE-MTL	0.299	0.052	0.695	0.478	0.381
12	GPR	0.343	0.575	0.830	-0.229	0.380
13	Naive-Ensemble	0.275	0.066	0.692	0.388	0.355
14	Transformer	0.334	0.113	0.711	0.086	0.311
15	AutoDLFormer	0.145	0.070	0.711	0.152	0.269
16	LSTM	0.100	0.124	0.570	0.115	0.227

S4.6 Locked hybrid robustness

seeds 42/43/44 give mean pooled R2 0.693/0.689/0.693 (mean 0.691 ± 0.002), and all three runs remain above the frozen MLP-sklearn reference. the locked hybrid reaches mean pooled R2 0.688 without the five test-condition inputs, 0.065 above the best retrained reference. the locked hybrid obtains LORO R2 0.429/0.592/0.897/0.681 (mean 0.650), 0.003 below the best LORO reference.

Table S10. Main, no-test-condition, LORO and three-seed confirmation of the locked hybrid.

Setting	Hybrid mean R2	Reference mean R2	Difference	Rank
Main grouped CV	0.693	0.656	0.037	1/16
No test-condition inputs	0.688	0.623	0.065	1/16
Leave one recipe out	0.650	0.653	-0.003	2/5
Three-seed mean	0.691	0.656	0.035	1/16

S5. Inference and per-design diagnostics

S5.1 Locked hybrid versus the strongest reference

taskwise R2 differences from MLP-sklearn are -0.009/-0.049/-0.006/+0.211; 0 of 4 survives Holm correction, and all four 95% recipe-bootstrap intervals include zero.

Table S11. Taskwise recipe-bootstrap comparison of the locked hybrid with MLP-sklearn.

task	delta_r2	ci_low	ci_high	p_bootstrap	n_boot	p_holm
Penetration	-0.009	-0.223	0.123	0.833	5000	1.000
Softening Point	-0.049	-0.223	0.018	0.216	5000	0.672
Ductility	-0.006	-0.096	0.046	0.802	5000	1.000
Rutting Factor	0.211	-1.204	0.377	0.168	5000	0.672

S5.2 Original-model paired comparisons with confidence intervals

56 comparisons were made; 7 survive task-wise Holm correction under the recipe-level bootstrap and 0 under the fold-level Wilcoxon test used in the submitted version. the 95 per cent interval of the R2 difference covers zero in 40 of 56 comparisons.

Table S12. Difference in the pooled coefficient of determination between TA-MoE-MTL and each baseline, with 95 per cent confidence intervals obtained by resampling mix designs, together with Holm-corrected bootstrap and fold-level Wilcoxon p-values.

baseline	task	delta_r2	ci_low	ci_high	p_bootstrap	n_boot	p_wilcoxon_fold	p_bootstrap_holm	p_wilcoxon_holm
AutoDLFormer	Penetration	0.155	0.057	0.852	0.004	2000	0.312	0.052	1.000
AutoDLFormer	Softening Point	-0.018	-0.279	0.174	0.787	2000	1.000	1.000	1.000
AutoDLFormer	Ductility	-0.016	-0.351	0.346	0.988	2000	0.285	1.000	1.000
AutoDLFormer	Rutting Factor	0.326	-0.041	0.606	0.569	2000	1.000	1.000	1.000
GRU	Penetration	-0.191	-0.799	-0.036	0.020	2000	0.438	0.200	1.000
GRU	Softening Point	0.042	-0.036	0.129	0.214	2000	0.188	1.000	1.000
GRU	Ductility	-0.105	-0.552	0.169	0.416	2000	1.000	1.000	1.000
GRU	Rutting Factor	0.033	-0.045	0.159	0.544	2000	0.812	1.000	1.000
LSTM	Penetration	0.200	0.086	0.853	0.001	2000	0.312	0.014	1.000
LSTM	Softening Point	-0.072	-0.362	0.207	0.493	2000	0.625	1.000	1.000
LSTM	Ductility	0.125	-0.182	0.552	0.342	2000	1.000	1.000	1.000
LSTM	Rutting Factor	0.362	-0.323	0.645	0.233	2000	0.062	1.000	0.875
MLP	Penetration	-0.242	-0.723	-0.058	0.027	2000	0.062	0.243	0.875
MLP	Softening Point	-0.089	-0.261	0.078	0.199	2000	0.438	1.000	1.000
MLP	Ductility	0.019	-0.373	0.241	0.860	2000	0.285	1.000	1.000
MLP	Rutting Factor	0.135	-0.132	0.359	0.075	2000	0.625	0.750	1.000
Transformer	Penetration	-0.035	-0.328	0.250	0.632	2000	0.625	1.000	1.000
Transformer	Softening Point	-0.061	-0.386	0.263	0.611	2000	0.812	1.000	1.000
Transformer	Ductility	-0.016	-0.375	0.537	0.991	2000	0.109	1.000	1.000
Transformer	Rutting Factor	0.392	-0.323	0.580	0.162	2000	0.125	1.000	1.000
Mamba	Penetration	-0.229	-0.959	-0.026	0.037	2000	0.125	0.296	1.000
Mamba	Softening Point	-0.136	-0.384	0.182	0.282	2000	0.812	1.000	1.000
Mamba	Ductility	0.027	-0.340	0.799	0.734	2000	0.109	1.000	1.000

baseline	task	delta_r2	ci_low	ci_high	p_bootstrap	n_boot	p_wilcoxon_fold	p_bootstrap_holm	p_wilcoxon_holm
Mamba	Rutting Factor	0.198	-0.128	0.269	0.111	2000	0.125	0.999	1.000
Naive-Ensemble	Penetration	0.024	-0.221	0.201	0.711	2000	1.000	1.000	1.000
Naive-Ensemble	Softening Point	-0.014	-0.101	0.241	0.845	2000	0.625	1.000	1.000
Naive-Ensemble	Ductility	0.003	-0.113	0.222	0.977	2000	1.000	1.000	1.000
Naive-Ensemble	Rutting Factor	0.090	-0.328	0.296	0.674	2000	0.312	1.000	1.000
ElasticNet	Penetration	-0.194	-0.771	0.378	0.271	2000	0.438	1.000	1.000
ElasticNet	Softening Point	-0.614	-1.258	-0.293	0.000	2000	0.062	0.000	0.875
ElasticNet	Ductility	-0.147	-0.806	0.122	0.283	2000	0.285	1.000	1.000
ElasticNet	Rutting Factor	0.177	-0.203	0.271	0.189	2000	1.000	1.000	1.000
RandomForest	Penetration	-0.276	-1.060	0.040	0.071	2000	0.312	0.426	1.000
RandomForest	Softening Point	-0.610	-1.288	-0.258	0.000	2000	0.125	0.000	1.000
RandomForest	Ductility	-0.063	-0.467	0.298	0.703	2000	0.593	1.000	1.000
RandomForest	Rutting Factor	0.157	-0.004	0.267	0.060	2000	1.000	0.660	1.000
SVR-RBF	Penetration	-0.313	-1.247	-0.090	0.010	2000	0.312	0.110	1.000
SVR-RBF	Softening Point	-0.570	-1.118	-0.255	0.000	2000	0.062	0.000	0.875
SVR-RBF	Ductility	0.025	-0.401	0.868	0.886	2000	0.593	1.000	1.000
SVR-RBF	Rutting Factor	0.350	-0.253	2.273	0.478	2000	1.000	1.000	1.000
GPR	Penetration	-0.044	-0.511	1.190	0.805	2000	1.000	1.000	1.000
GPR	Softening Point	-0.523	-1.095	-0.158	0.008	2000	1.000	0.072	1.000
GPR	Ductility	-0.136	-0.859	0.205	0.387	2000	1.000	1.000	1.000
GPR	Rutting Factor	0.707	0.342	0.933	0.003	2000	0.312	0.042	1.000
XGBoost	Penetration	-0.126	-0.740	0.420	0.494	2000	0.625	1.000	1.000
XGBoost	Softening Point	-0.420	-1.123	0.183	0.133	2000	0.812	1.000	1.000
XGBoost	Ductility	-0.129	-0.986	0.347	0.438	2000	0.593	1.000	1.000
XGBoost	Rutting Factor	0.249	0.014	0.452	0.031	2000	1.000	0.403	1.000
LightGBM	Penetration	-0.320	-1.423	-0.083	0.005	2000	0.125	0.060	1.000
LightGBM	Softening Point	-0.601	-1.142	-0.320	0.000	2000	0.062	0.000	0.875
LightGBM	Ductility	-0.153	-0.544	0.049	0.148	2000	1.000	1.000	1.000
LightGBM	Rutting Factor	0.379	0.100	1.973	0.034	2000	1.000	0.408	1.000
MLP-sklearn	Penetration	-0.322	-1.195	0.015	0.055	2000	0.312	0.385	1.000
MLP-sklearn	Softening Point	-0.591	-1.138	-0.283	0.000	2000	0.062	0.000	0.875
MLP-sklearn	Ductility	-0.190	-0.880	0.054	0.158	2000	0.285	1.000	1.000
MLP-sklearn	Rutting Factor	0.003	-0.064	0.104	0.812	2000	0.812	1.000	1.000

S5.3 Leave-one-recipe-out errors

leave-one-recipe-out over all 20 designs gives pooled R2 0.670/0.617/0.818/0.507 for penetration, softening point, ductility and rutting factor respectively.

Table S13. Mean absolute error per mix design under leave-one-recipe-out validation, in the physical unit of each target.

Recipe	Pen	SP	Duc	Rut
R01	2.254	3.714		6.818
R02	7.473	2.144		0.708
R03	1.206	2.252		1.098
R04	0.901	1.505		0.286
R05	0.652	2.838		2.907
R06	1.548	2.112		2.135
R07	7.066	3.174		1.243
R08	1.018	0.052		1.531
R09	5.583	4.162		1.190
R10	6.020	2.049		1.008
R11	2.098	1.512		7.098
R12	0.750	0.167		1.666
R13	6.818	18.032	65.300	64.900
R14	10.636	2.081	104.712	266.937
R15	9.148	5.061	19.037	324.282
R16	4.801	5.096	9.366	1281.2
R17	21.227	7.206	1.831	946.325
R18	10.744	3.729	1.759	7.695
R19	0.916	0.544	2.679	9.823
R20	9.746	2.471	2.974	11.683

S5.4 Residual diagnostics

These statistics complement the recipe-coloured observed-versus-predicted plot and the per-recipe leave-one-out errors in Figures 10 and 11 of the main text.

Table S14. Out-of-fold residual diagnostics for TA-MoE-MTL under the nested protocol, including bias, dispersion, tail error and skewness.

task	n	recipes	bias	median	sd	MAE	RMSE	p95_abs	skew	corr_MAE_distance
Penetration	221	20	3.700	4.460	12.807	11.371	13.303	23.186	-0.521	-0.016
Softening Point	221	20	-0.853	-0.091	10.034	7.112	10.047	23.001	-0.051	0.143
Ductility	155	8	15.045	9.732	55.038	42.692	56.886	101.107	0.462	0.094
Rutting Factor	221	20	-167.344	5.523	496.137	259.884	522.534	1358.0	-1.466	-0.046

S5.5 Learning-curve runs

4 designs → -1.783 ± 0.474; 8 designs → -40.597 ± 66.892; 12 designs → -0.351 ± 0.675; 16 designs → 0.390 ± 0.194

Table S15. Individual learning-curve runs: number of training designs, draw index, number of training rows, selected epoch and held-out performance.

n_train_recipes	seed	n_train_rows	selected_epoch	Pen	SP	Duc	Rut	mean_r2
4.000	0.000	62.000	15.000	-2.269	-2.717	0.000	-1.841	-1.707
8.000	0.000	102.000	59.000	0.876	-1.098	0.000	-471.108	-117.832
12.000	0.000	145.000	23.000	-0.963	0.762	0.000	0.324	0.031
16.000	0.000	181.000	60.000	0.960	0.993	0.000	0.389	0.586
4.000	1.000	40.000	27.000	-4.506	-3.719	0.000	-0.934	-2.289
8.000	1.000	91.000	3.000	-4.828	-5.414	0.000	-0.928	-2.793
12.000	1.000	141.000	8.000	0.089	-0.541	0.000	0.640	0.047

n_train_recipes	seed	n_train_rows	selected_epoch	Pen	SP	Duc	Rut	mean_r2
16.000	1.000	188.000	89.000	0.197	0.640	0.000	-0.044	0.198
4.000	2.000	48.000	9.000	-2.543	-0.971	-1.345	-0.549	-1.352
8.000	2.000	86.000	64.000	-6.451	0.407	0.932	0.443	-1.167
12.000	2.000	124.000	27.000	-1.236	-4.699	0.973	0.439	-1.131
16.000	2.000	156.000	175.000	0.298	0.624	0.527	0.094	0.386

S6. Attribution

S6.1 Surrogate fidelity

The two columns answer different questions. In-domain fidelity determines whether the attributions describe the network at all; fidelity on unseen designs determines whether they may be read as mechanism for new formulations. the coefficient of determination between surrogate and network is 0.998/0.985/1.000/1.000 for penetration, softening point, ductility and the rutting factor respectively, whereas the corresponding values are -16.30/-3.92/-908.72/-9.39.

Table S16. Fidelity of the attribution surrogate under random splitting and under mix-design-grouped splitting.

Target	Fidelity, in-domain	Fidelity, unseen designs	Attribution reported	Mechanistic transfer supported
Penetration	0.998	-16.299	yes	no
Softening Point	0.985	-3.917	yes	no
Ductility	1.000	-908.721	yes	no
Rutting Factor	1.000	-9.389	yes	no

S6.2 Attribution by physical factor

Penetration: Rubber dosage 65 per cent, Shear processing 14 per cent; Softening Point: Rubber dosage 47 per cent, Shear processing 27 per cent; Ductility: Rubber dosage 92 per cent, Shear processing 8 per cent; Rutting Factor: Rubber dosage 84 per cent, Shear processing 11 per cent.

Table S17. Share of total attribution carried by each physical factor, per target.

task	factor	share
Penetration	Rubber dosage	0.647
Penetration	Shear processing	0.137
Penetration	Rubber particle size	0.113
Penetration	WMA chemistry	0.081
Penetration	Test condition	0.013
Penetration	Base binder	0.009
Softening Point	Rubber dosage	0.471
Softening Point	Shear processing	0.274
Softening Point	WMA chemistry	0.172
Softening Point	Rubber particle size	0.045
Softening Point	Test condition	0.026
Softening Point	Base binder	0.012
Ductility	Rubber dosage	0.921
Ductility	Shear processing	0.079
Rutting Factor	Rubber dosage	0.841
Rutting Factor	Shear processing	0.110
Rutting Factor	Base binder	0.021
Rutting Factor	Rubber particle size	0.014
Rutting Factor	WMA chemistry	0.010
Rutting Factor	Test condition	0.003
